# Atypical functional hierarchy contributed to the tinnitus symptoms in patients with vestibular schwannoma

**DOI:** 10.3389/fnins.2023.1084270

**Published:** 2023-02-17

**Authors:** Jiaji Lin, Na You, Xiaolong Li, Jiayu Huang, Haoxuan Lu, Jianxing Hu, Jun Zhang, Xin Lou

**Affiliations:** ^1^Department of Radiology, Chinese People’s Liberation Army General Hospital/Medical School of Chinese People’s Liberation Army, Beijing, China; ^2^Department of Neurosurgery, Chinese People’s Liberation Army General Hospital/Medical School of Chinese People’s Liberation Army, Beijing, China

**Keywords:** functional gradient, tinnitus, tinnitus handicap inventory, visual analog scale, vestibular schwannoma

## Abstract

**Objective:**

Tinnitus is frequently found in patients with vestibular schwannoma (VS), but its underlying mechanisms are currently unclear.

**Methods:**

Both preoperative (VS_*pre*_) and postoperative (VS_*post*_) functional MR images were collected from 32 patients with unilateral VS and matched healthy controls (HCs). Connectome gradients were generated for the identification of altered regions and perturbed gradient distances. Tinnitus measurements were conducted for predictive analysis with neuroimaging–genetic integration analysis.

**Results:**

There were 56.25% of preoperative patients and 65.63% of postoperative patients suffering from ipsilateral tinnitus, respectively. No relevant factors were identified including basic demographics info, hearing performances, tumor features, and surgical approaches. Functional gradient analysis confirmed atypical functional features of visual areas in VS_*pre*_ were rescued after tumor resection, while the gradient performance in the postcentral gyrus continues to maintain (VS_*post*_ vs. HC : *P* = 0.016). The gradient features of the postcentral gyrus were not only significantly decreased in patients with tinnitus (*P*_*FDR*_ = 0.022), but also significantly correlated with tinnitus handicap inventory (THI) score (*r* = −0.30, *P* = 0.013), THI level (*r* = −0.31, *P* = 0.010), and visual analog scale (VAS) rating (*r* = −0.31, *P* = 0.0093), which could be used to predict VAS rating in the linear model. Neuropathophysiological features linked to the tinnitus gradient framework were linked to Ribosome dysfunction and oxidative phosphorylation.

**Conclusion:**

Altered functional plasticity in the central nervous system is involved in the maintenance of VS tinnitus.

## 1. Introduction

Subjective tinnitus is a common symptom of ear dysfunction and is frequently found in patients with vestibular schwannoma (VS) (Andersson et al., 1997). About 10% of patients with VS have tinnitus as the initial manifestation and 80% of patients develop tinnitus during the progression (Wang et al., 2020). This tinnitus persists as the high pitch of “cicada chirping” or “buzzing sound,” which not only affects the normal hearing of patients but also induces sleep disorders, anxiety, and depression (Chovanec et al., 2015). Although a large body of literature exists on the possible causes and remedies for VS tinnitus, especially peripheral mechanisms (compression, invasion, ischemia, etc.), surgical resection of the peripheral tumor is found not as an effective treatment for VS tinnitus and it may even cause new tinnitus (Chovanec et al., 2015). Therefore, it is often suspected that functional plasticity alteration in the central nervous system may be involved in the occurrence and maintenance of VS tinnitus.

The brain function activity, including hearing sensory, strictly follows hierarchical principles for functional requirements, providing the basic framework for information exchange and processing across modalities (Margulies et al., 2016). Multi-level manifold representations have been summarized to illustrate this functional fashion, for example, progressive motor information conversion in the rostral-caudal gradient of the prefrontal cortex (Badre and D’Esposito, 2009), and abstraction of visual information conversion in the ventral-dorsal gradient of the temporal cortex (Mishkin and Ungerleider, 1982). Convergent evidence from connectome features, genetical, profiles, and microstructural signatures further demonstrate the broad biological influence of this hierarchical architecture (Huntenburg et al., 2018). The consequences of functional hierarchy disruption are systemic, not only related to the sensory specialization (Huntenburg et al., 2018), but also to cognitive performance and motor behaviors (Wang et al., 2021), which are also frequently deficient in various nervous diseases, including Austin (Hong et al., 2019), ischemic stroke (Bayrak et al., 2019), and epilepsy (Meng et al., 2021). Further research on tinnitus from the perspective of hierarchical principles may help us to better understand the regulation and maintenance of abnormal hearing perception.

To this end, our present study collected imaging data from patients with VS for functional hierarchy analysis. By characterizing global and regional alteration signatures, we also try to explore their possibility for tinnitus symptom prediction. In addition, we also hypothesized and performed neuroimaging–genetic integration analysis to identify potential neuropathophysiological susceptibilities.

## 2. Materials and methods

### 2.1. Experimental design and grouping

This is a retrospective study to study the neuroplasticity mechanisms associated with VS in Chinese PLA General Hospital from January 2019 to April 2022. It was approved by the institutional review board and the independent scientific advisory committee at the Chinese PLA General Hospital. The data are anonymous, and the requirement for informed consent was, therefore, waived. The data for *post hoc* retrospective analysis can be divided into two parts: (1) We reviewed all patients with VS who underwent unilateral resection *via* retrosigmoid approach from January 2019 to April 2022, and 110 cases with preoperative and postoperative resting-state functional MRI (rs-fMRI) imaging were screened out. The tumor diagnosis of these patients was based on WHO criteria and was confirmed by postoperative pathological examination. The main exclusion criteria included any other abnormality or disease, a history of alcohol or illegal substance abuse, and a history of other brain surgery or neuromodulation interventions. In the end, the data of a total of 32 patients were finally included in the present study. The data of patients with VS were divided into a pre-operative group (VS_*pre*_) and a post-operative group (VS_*post*_). Moreover, the data of the patients were also divided into a group with tinnitus (VS_+*tinnitus*_) and a group without tinnitus (VS_−*tinnitus*_) according to tinnitus symptoms. (2) The data of healthy subjects. All the health controls accepted health examinations in the Chinese PLA General Hospital during the same period. The inclusion criteria included being older than 18 years and having functional imaging data. The main exclusion criteria include any other abnormality or disease, a history of alcohol or illegal substance abuse, MRI contraindications or intolerance, and any history of surgery or invasive intervention. Thirty-two of them matching the sex, age, and education of patients with VS were selected as healthy controls, and the image data of another 32 healthy subjects were used as an unrelated healthy dataset for gradients’ alignment.

### 2.2. MRI data acquisition and preprocessing

MRI data were acquired by a 3T MRI scanner (Discovery 750, GE Healthcare, USA). Participants were asked to keep their eyes closed, relax, and wear earplugs to reduce noise, and foam pads were placed around the head to minimize head motion during MRI acquisition. The rs-fMRI data were collected based on the echo-planar imaging sequence: TE = 30 msec, TR = 2,000 msec, FA = 90°, FOV = 240 mm × 240 mm, matrix = 64 × 64, slice thickness = 3.5 mm, slice gap = 0.5 mm, and volume = 180. Two senior radiologists independently reviewed the sequences and checked the quality of the images.

The rs-fMRI data were preprocessed using Graph Theoretical Network Analysis (GRETNA) toolbox (Wang et al., 2015). Briefly, the preprocess consisted of the following steps: (1) the Dicom images were converted to NIfTI format; (2) the top five time points were removed; (3) the images underwent slice timing and correction for head motion; (4) all images were spatially normalized with the standard Montreal Neurological Institute (MNI) space; (5) spatial smooth was performed using a 4 mm × 4 mm × 4 mm full with a half-maximum Gaussian kernel; and (6) linear trend removal and nuisance covariate regression was performed with nuisance variables including Friston 24 parameter correction, white matter signal, and cerebrospinal fluid signal. Bandpass filtering was 0.01–0.1 Hz.

### 2.3. Functional connectome and gradient analysis

In order to explore whole-brain gradients alteration, we merged multiple intrinsic functional connectivity-based atlases, including cortical parcellations (Schaefer et al., 2018), cerebellar parcellations (Buckner et al., 2011), striatal parcellations (Choi et al., 2012), and thalamic parcellations (Horn and Kühn, 2015). There were three subcortical regions of interest (ROIs) that were discarded during 3 mm × 3 mm × 3 mm down-sampling due to small sizes. The final remaining 1,039 ROIs have corresponding functional community annotation, including Visual (Vis), Somatomotor (SM), Dorsal Attention (DA), Ventral Attention (VA), Limbic (Lim), Frontoparietal (FP), and Default Mode Network (DMN) (Yeo et al., 2011). Pearson correlation coefficients were computed for each pair of brain regions as the functional connectome. Detailed information about this brain atlas could be found in Supplementary Table 1 or https://github.com/louxin-lab.

The functional connectome was then *z*-transformed and the top 10% thresholded, and the cosine similarity matrix was calculated to capture similarity in connectivity profiles (Vos de Wael et al., 2020). Principal component analysis (PCA), the most reproducible dimensionality reduction algorithm for gradient framework, was applied to identify primary gradient components for the majority of connectome variance (Hong et al., 2020). A group-level gradient component template was generated from an average connectivity matrix based on unrelated health datasets as mentioned earlier, and we performed Procrustes rotation to align components to the template (Vos de Wael et al., 2020). As with most gradient studies, we mainly focused on the primary two components (Gradient-1 and Gradient-2) as they explained the majority of the total variance. These components, initially defined in connectivity space, were then mapped back onto the ROIs to visualize macroscale transitions in overall connectivity patterns. To analyze the functional distance alteration between ROIs, we used the primary two gradients to calculate the Euclidean distance in the functional hierarchical architecture. As for statistical analysis, independent and paired *t*-tests were applied for VS vs. healthy controls (HCs) comparison or VS_*pre*_ vs. VS_*post*_ comparison, respectively, with False Discovery Rate (FDR) correction.

### 2.4. Tinnitus status evaluation and symptom prediction

During the rs-fMRI follow-up, we mainly used tinnitus handicap inventory (THI) and the visual analog scale (VAS) to evaluate the ipsilateral tinnitus of patients with VS. The THI is the most commonly used questionnaire of a 25-item to measure tinnitus-produced handicap. Patients can answer “no,” “sometimes,” or “yes” to each item (corresponding to scores 0, 2, and 4) (Newman et al., 1996). The global score is used as an index of the severity of tinnitus, with a grading included between 0 and 100. According to the THI score, it could be divided into different THI levels, including Level-1 (very mild, 1–16 points), Level-2 (mild, 18–36 points), Level-3 (moderate, 38–56 points), Level-4 (severe, 58–76 points), and Level-5 (catastrophic, 78–100 points). The change in the patient’s tinnitus status (improve or worsen) is mainly determined by the alteration of the THI score (increase or decrease). The VAS of tinnitus intensity was used with the left and right extremes labeled “very faint” and “very loud,” respectively (Raj-Koziak et al., 2018). Patients were required to give a first estimation of the intensity of their tinnitus at the beginning of the testing session before any sound was presented, and then be asked to use this VAS only when partial residual inhibition was obtained in a condition.

We also tried to apply gradient features for tinnitus measurements (VAS rating, THI score, and THI level) using stepwise linear models with leave-one-out cross-validation (LOOSWR). The gradient features were extracted from the brain regions with the most significant alteration and were fit into linear regression with adjustment of the subject’s gender, age, and constant. At each iteration, the predictive performance for the tinnitus measurements was assessed and only *P* < 0.05 was considered statistically significant when making predictions. Using both forward and backward iterations, over 98% of iterations (gradient predictors were significant and entered a model) were reported as reliable features. The Pearson correlation coefficient and the mean absolute error (MAE) between the predicted value and the actual measurements were used to evaluate the prediction effect.

### 2.5. Neuropathophysiological features analysis of gradient alteration

The Allen Human Brain Atlas (AHBA) was introduced to study neuropathophysiological features underlying gradient framework alteration subjected to tinnitus. AHBA comprises available transcriptomic dataset post-mortem samples from six adult male donors (Markello et al., 2021). Detailed descriptions of the methods of whole genome microarray analysis could be found in Allen Institute for Brain Science technical white paper. In the present study, 20,737 genes of 1,039 regions were extracted as a 20,737 × 1,039 matrix. The framework alteration was established through a *t*-test between the pre- and post-operative global gradient features. We then used partial least squares regression (PLSR) to investigate the fundamental relationships between the framework alteration and gene expression with 10,000 permutation tests. The PLS1 is defined as the most strongly correlated spatial signature during the linear analysis of the gene expression weights. The gene ranked list according to the PLS1 performance was fitted into the WebGestalt to identify Gene Ontology (GO) and the Kyoto Encyclopedia of Genes and Genomes (KEGG) enrichment by Gene Set Enrichment Analysis (GSEA) (Liao et al., 2019).

## 3. Results

### 3.1. Clinical features impacting postoperative VS tinnitus

A total of 32 patients with unilateral VS were finally included in the present study. The mean age of all patients was 46.44 ± 12.13 years, and 12 of these patients were male. Among the patients, 65.63% of patients had preoperative hearing impairment of varying degrees with an average tumor size of 2.44 ± 1.21 cm. As for tinnitus symptoms, there were 56.25% of preoperative patients and 65.63% of postoperative patients suffered from subjective tinnitus. The preoperative THI scores, THI level, and VAS rating of patients with tinnitus were 22.11 ± 18.38, 1.83 ± 1.02, and 4.33 ± 2.14, respectively, while the postoperative THI scores, THI level, and VAS rating were 20.95 ± 17.54, 1.71 ± 0.91, and 4.10 ± 1.90, respectively. The detailed cohort info could be found in Table 1.

**TABLE 1:** Characteristics of patients with vestibular schwannoma (VS) (*n* = 32).

Characteristics	Number (%)		*P*-value*
	**With tinnitus**	**Without tinnitus**	
Gender			0.718
Male	6 (33.3)	6 (42.9)	
Female	12 (66.7)	8 (57.1)	
Age			0.722
≥50	8 (44.4)	8 (57.1)	
<50	10 (55.6)	6 (42.9)	
Side			1.0
Left	9 (50.0)	7 (50.0)	
Right	9 (50.0)	7 (50.0)	
AAO-HNS			/
Class A	4 (22.2)	5 (35.7)	
Class B Class C Class D	5 (27.8)) 2 (11.1) 7 (38.9)	3 (21.4) 0 (0.0) 6 (42.8)	
Preoperative hearing			0.735
Serviceable hearing	9 (50.0)	8 (57.1)	
(Class A and B)	9 (50.0)	6 (42.9)	
Unserviceable hearing (Class C and D)			
Tumor size			0.252
>3 cm	3 (16.7)	5 (35.7)	
≤3 cm	15 (83.3)	9 (64.3)	
Cochlear nerve			0.613
Preserved	3 (16.7)	1 (7.1)	
Cut	15 (83.3)	13 (92.9)	
Degree of resection			0.132
Total resection	15 (83.3)	8 (57.1)	
Subtotal resection	3 (16.7)	6 (42.9)	
Tumor nature			0.473
Solid	12 (66.6)	7 (50.0)	
Non-solid	6 (27.8)	7 (7.1)	

*Fisher’s exact test.

**FIGURE 1:**
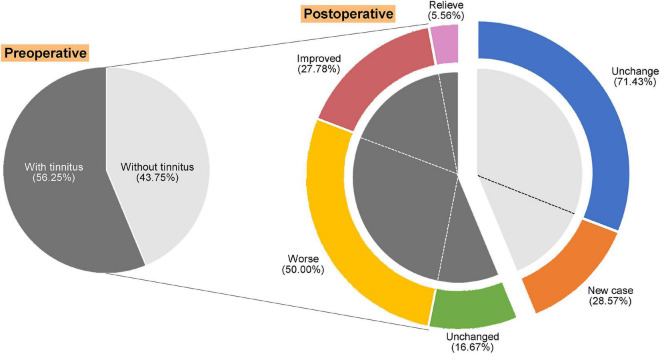
Alteration and distribution of tinnitus symptoms in patients with vestibular schwannoma (VS) after surgery.

The change in the patient’s tinnitus status (improve or worsen) is mainly determined by the alteration of THI score (Figure 1). After VS resection, there was only one (5.56%) that was totally resolved, five (27.78%) improved, and 13 (50.00%) worse. It was interesting to note that 28.57% of the patients with no preoperative tinnitus developed postoperative tinnitus. To analyze the factors affecting postoperative tinnitus, we divided patients with tinnitus into two groups: *Resolved + Improved* (33.34%) *and Unchanged + Worse* (66.66%). We compared the differences between the two groups in terms of demographics info, preoperative hearing, tumor features (tumor size, occupation of internal auditory canal, tumor nature, and cerebellum brain stem extrusion), and surgery approach (cochlear nerve and degree of resection). The results showed that none of these factors had a significant effect on postoperative tinnitus alteration (Table 2).

**TABLE 2:** Factors affecting postoperative tinnitus alteration (*n* = 22).

Characteristics	Change of tinnitus		*P* value*
	**Resolved + improved**	**Unchanged + worse**	
Gender (Male/Female)	4/2	4/12	0.137
Age (≥50/<50)	3/3	6/10	0.655
Preoperative hearing (Class A and B/Class C and D)	2/4	11/5	0.178
Tumor size (≤3 cm/>3 cm)	5/1	14/2	1.000
Occupation of internal auditory canal (≤1 cm/>1 cm)	6/0	10/6	0.133
Tumor nature (solid/non-solid)	4/2	12/4	1.000
Cerebellum brain stem extrusion (+/−)	2/4	11/5	0.178
Cochlear nerve (+/−)	0/6	4/12	0.541
Degree of resection (total resection/subtotal resection)	5/1	13/3	1.000

*Fisher’s exact test.

### 3.2. Perturbed functional architectures of visual areas were rescued after VS resection

**FIGURE 2:**
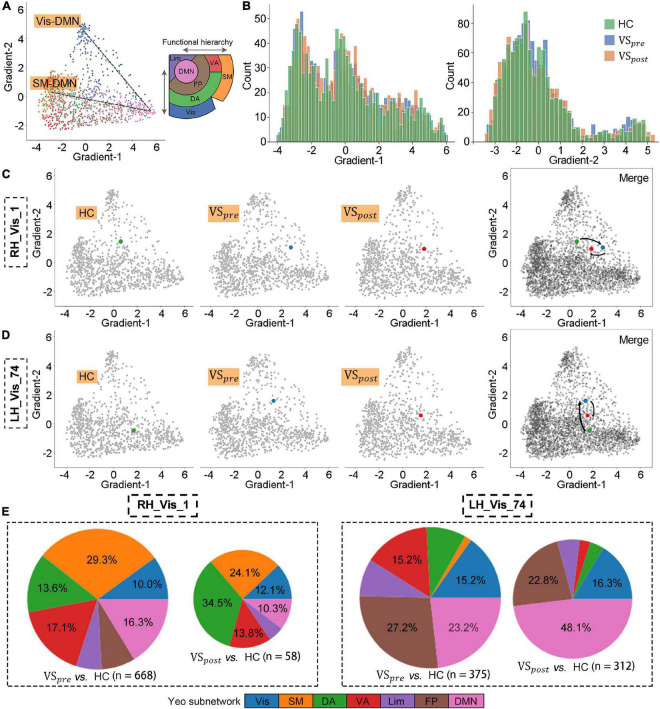
Perturbed functional architectures of visual areas were rescued after vestibular schwannoma (VS) resection. **(A)** Gradient-1 mainly showed a gradual Visual-Default Mode Network (Vis-DMN) axis of connectivity variations, while Gradient-2 showed a gradual SM-DMN axis of connectivity variations in healthy adults. **(B)** Gradient distribution in each group. **(C,D)** Regional gradients signature of *RH_Vis_1* and *LH_Vis_24*. **(E)** The number of brain regions whose gradient distance is altered from the *RH_Vis_1* or *LH_Vis_24*, and their distribution in Yeo functional communities.

**FIGURE 3:**
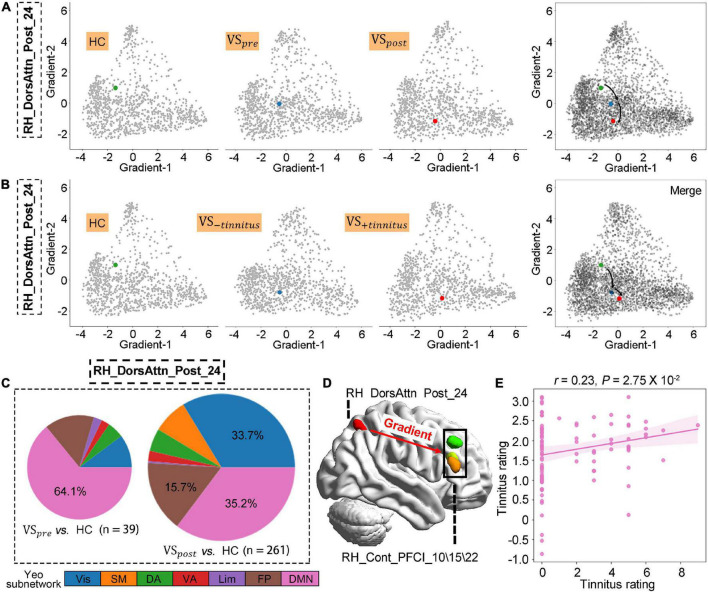
Altered postcentral gradient features in patients with vestibular schwannoma (VS) reflected tinnitus symptoms. **(A,B)** Regional gradients signature of *RH_DorsAtten_post_24* based on tumor resection or tinnitus symptom. **(C)** The number of brain regions whose gradient distance is altered from the *RH_DorsAtten_post_24* around tumor resection, and their distribution in Yeo functional communities. **(D)** Gradient distances were only significantly increased from *RH_DorsAtten_post_24* with *RH_Cont_PFCI_10/15/22* in VS_+*tinnitus*_ vs. healthy controls (HCs). **(E)** Gradient-2 features of *RH_DorsAtten_post_24* were also included as independent variables in leave-one-out cross-validation (LOOSWR) models for tinnitus predication with adjustment of gender and age in patients with VS.

The primary Gradient-1 and Gradient-2 accounted for a total variance of 69% in VS_*pre*_ (Figure 2A). No statistical difference was identified for variance explanation of global functional connectome in the comparison of VS_*pre*_ vs. HCs or VS_*pre*_ vs. VS_*post*_ (Figure 2B). We then investigated regional alteration. Independent *t*-test revealed that Gradient-1 features of the No. 501 ROI of the visual cortex on the right hemisphere (known as *RH_Vis_1* in Schaefer 1,000 Parcels 7 Networks atlas, MNI: *x* = 33, *y* = −36, *z* = −23, VS_*pre*_ vs. HCs: *t*-test *P*_*FDR*_ = 0.004) and Gradient-2 features of the No. 24 ROI of the visual cortex on the left hemisphere (known as *LH_Vis_24* in Schaefer 1,000 Parcels 7 Networks atlas, MNI: *x* = −34, *y* = −92, *z* = −9, VS_*pre*_ vs. HCs: *t*-test *P*_*FDR*_ = 0.042) were found abnormal in VS_*pre*_ compared with matched HCs (Figures 2C, D). Both altered features of *RH_Vis_1* and *LH_Vis_24* were found rescued after tumor resection (VS_*post*_ vs. HCs: both *t*-test *P*_*FDR*_ > 0.05) (Figures 2C, D). No gradient features were found significantly altered in any of the subcortical structures (cerebellum, thalamus, and striatum). Moreover, it was found Gradient-2 features of the No. 708 ROI of the posterior cortex of dorsal attention on the right hemisphere (known as *RH_DorsAttn_Post_24* in Schaefer 1,000 Parcels 7 Networks atlas, MNI: *x* = 29, *y* = −64, *z* = 52) were significantly decreased both preoperatively and postoperatively (VS_*pre*_ vs. HCs: t-test *P*_*FDR*_ > 0.05; VS_*post*_ vs. HCs: *t*-test *P*_*FDR*_ = 0.016) (Refer to Result section “3.3. Postcentral gradient features in patients with VS reflected tinnitus symptoms”; Figure 3A). These results were robust and virtually identical when controlling for confounding features of rs-fMRI processing, including connectivity matrix thresholding (10–20%; van Wijk et al., 2010; Supplementary Figure 1). Moreover, we obtained similar results with the exclusion of the patients with preserved cochlear nerves (Supplementary Figure 1).

According to functional hierarchy theory, we analyzed the gradient distances related to altered regions. With the threshold of *t*-test *P*_*FDR*_ < 0.05, the gradient distances from *RH_Vis_1* to the other 668 nodes were significantly altered in the VS_*pre*_ compared with HCs, which mainly were restored in VS_*post*_ (58 nodes, Figure 2E). As for *LH_Vis_24*, there were 375 altered gradient distances in the VS_*pre*_ compared with HCs, and still 312 altered gradient distances in the VS_*post*_ (Figure 2E). Gradient distances from *LH_Vis_24* to DMN regions were significantly increased after VS resection (23.2% in VS_*pre*_ vs. 48.1% in VS_*post*_).

### 3.3. Postcentral gradient features in patients with VS reflected tinnitus symptoms

The VS occurrence initialed the gradient features alteration of *RH_DorsAtten_post_24*, and these changes were further exacerbated after tumor resection (VS_*pre*_ vs. HCs: *t*-test *P*_*FDR*_ > 0.05; VS_*post*_ vs. HC : *t*-test *P*_*FDR*_ = 0.016; Figure 3A). To explore the potential link between gradient features and tinnitus symptoms, the VS data were regrouped into two clusters according to tinnitus status: (1) VS_+*tinnitus*_ group: 39 cases with tinnitus including 18 preoperative and 21 postoperative data and (2) VS_−*tinnitus*_ group: 25 cases without including 14 preoperative and 11 postoperative data. As for global gradient features, no statistical difference was identified for functional connectome variance in the comparison of VS_+*tinnitus*_ vs. VS_−*tinnitus*_ or VS_+*tinnitus*_ vs. HCs. As for regional alteration analysis, it was found that Gradient-2 features of *RH_DorsAtten_post_24* were also significantly decreased in VS_+*tinnitus*_ (VS_−*tinnitus*_ vs. HCs: *t*-test *P*_*FDR*_ > 0.05; VS_+*tinnitus*_ vs. HCs: *t*-test *P*_*FDR*_ = 0.022; Figure 3B).

We also analyzed the gradient distance alteration of *RH_DorsAtten_post_24* after tumor resection. Although there were only 39 nodes with significantly altered gradient distances from *RH_DorsAtten_post_24* in the VS_*pre*_ compared with HCs, there was a dramatical increase of 261 regions in VS_*post*_ with the threshold of *t*-test *P*_*FDR*_ < 0.05 (Figure 3C). Based on the Yeo subnetwork theory, it can be found that the altered gradient distance of *RH_DorsAtten_post_24* mainly connected to the brain regions within the DMN subnetwork (64.1% in VS_*pre*_ and 35.2% in VS_*post*_). Moreover, we compared the specific gradient distance features of the *RH_DorsAtten_post_24* in VS_+*tinnitus*_ or VS_−*tinnitus*_ compared with HCs. It could be found that gradient distances were only significantly increased from *RH_DorsAtten_post_24* with *RH_Cont_PFCI_10/15/22* in VS_+*tinnitus*_ vs. HCs (*t*-test *P*_*FDR*_ = 0.047/0.040/0.047, respectively; Figure 3D). All these gradient distances were found significantly correlated with tinnitus status evaluation (Table 3).

**TABLE 3:** Gradient distances alteration correlated with tinnitus status evaluation.

Gradient distances	Correlation coefficient*	*P* *
THI score		
*RH_DorsAtten_post_24–RH_Cont_PFCI_10*	−0.38	0.12 × 10^–3^
*RH_DorsAtten_post_24–RH_Cont_PFCI_15*	−0.40	0.65 × 10^–4^
*RH_DorsAtten_post_24–RH_Cont_PFCI_22*	−0.39	0.89 × 10^–4^
THI level		
*RH_DorsAtten_post_24–RH_Cont_PFCI_10*	−0.39	0.80 × 10^–4^
*RH_DorsAtten_post_24–RH_Cont_PFCI_15*	−0.40	0.56 × 10^–4^
*RH_DorsAtten_post_24–RH_Cont_PFCI_22*	−0.40	0.65 × 10^–4^
VAS rating		
*RH_DorsAtten_post_24–RH_Cont_PFCI_10*	−0.40	0.73 × 10^–4^
*RH_DorsAtten_post_24–RH_Cont_PFCI_15*	−0.42	0.35 × 10^–4^
*RH_DorsAtten_post_24–RH_Cont_PFCI_22*	−0.42	0.39 × 10^–4^

*Spearman correlation.

Gradient-2 features of *RH_DorsAtten_post_24* were used for spearman correlation with tinnitus status evaluation. It was found that Gradient-2 features of *RH_DorsAtten_post_24* were significantly correlated with THI score (*r* = −0.30, *P* = 0.013), THI level (*r* = −0.31, *P* = 0.010), and VAS rating (*r* = −0.31, *P* = 0.0093). Considering the tinnitus symptoms and the *RH_DorsAtten_post_24* features were both relatively independent of tumor resection, Gradient-2 features of *RH_DorsAtten_post_24* were selected as independent variables in LOOSWR models for tinnitus symptoms prediction (Figures 3D, E). With iterative repetition of 100% and predictive significance <0.05, it could be used to predict postoperative improvement of VAS rating (MAE = 1.94 ± 1.21, *r* = −0.23 and *P* = 0.028). We also tried to use gradient features to predict THI score or THI level improvement but found no significant results.

### 3.4. Genetic signature linked to VS tinnitus framework

**FIGURE 4:**
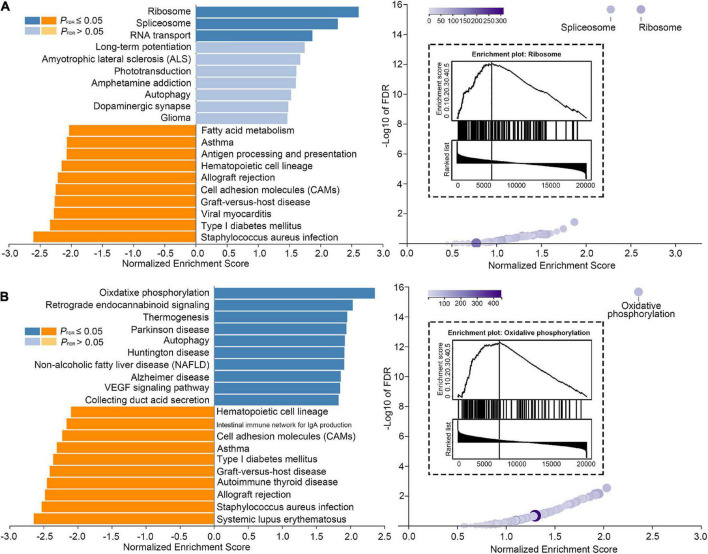
Genetic signature linked to tinnitus framework. **(A)** Significant results of Kyoto Encyclopedia of Genes and Genomes (KEGG) enrichment analysis performed for Gradient-1 alteration features. **(B)** Significant results of KEGG enrichment analysis performed for Gradient-2 alteration features.

We introduced AHBA to investigate featured genes underlying gradient, framework alteration related to tinnitus (VS_+*tinnitus*_ vs. HCs). The primary PLS component of featured genes accounted for 10.13% and 11.61% of the total variance significantly more than a random chance for Gradient-1 and Gradient-2 alteration features, respectively (*P* < 0.001). KEGG pathway analysis revealed that Gradient-1 featured genes were most significantly enriched in terms related to hsa03010 Ribosome (normalized enrichment score (NES) = 2.615, *P*_FDR_ < 0.001) with GO0070972 protein localization to the endoplasmic reticulum (NES = 2.379, *P*_FDR_ < 0.001), GO0006413 translational initiation (NES = 2.288, *P*_FDR_ < 0.001), GO0005840 ribosome (NES = 2.275, *P*_FDR_ < 0.001), and other GO annotations related to Ribosome (Figure 4A). Moreover, KEGG pathway analysis revealed that Gradient-2 featured genes were most significantly enriched in terms related to hsa00190 oxidative phosphorylation (NES = 2.361, *P*_FDR_ < 0.001) with GO0033108 mitochondrial respiratory chain complex assembly (NES = 2.270, *P*_FDR_ < 0.001), GO0044455 mitochondrial membrane part (NES = 2.400, *P*_FDR_ < 0.001), GO0070469 respiratory chain (NES = 2.256, *P*_FDR_ < 0.001), and other GO annotations related to oxidative phosphorylation (Figure 4B). Detailed gene enrichment results could be found in Supplementary Tables 2–9.

## 4. Discussion

Vestibular schwannoma is a benign tumor arising from the Schwann cell sheath investing cochleovestibular nerve; therefore, it is generally considered the prototype of a “retro-cochlear” sensorineural hearing abnormality. It is estimated that over 80% of patients with VS initially complain of hearing loss or tinnitus. Such hearing abnormality could be the result of various mechanisms (Stipkovits et al., 1998). Among them are direct compression of the cochlear nerve by tumor solids, cochlear dysfunction by ischemia, efferent system dysfunction following compression of the efferent fibers, biochemical changes in the inner ear, and cortical reorganization following tumor invasion of the cochlear nerve (Baguley et al., 2006). Another cause that has emerged as an interesting possible explanation of this symptom relates to a toxic substance produced by the tumor (Stipkovits et al., 1998; Stankovic et al., 2009). Although these peripheral mechanism hypotheses offer different explanations for hearing abnormality, they seem unable to explain the tinnitus characteristic of VS. No strong correlation has been not found between tinnitus symptoms and tumor characteristics, including tumor side, tumor size, internal auditory canal mass, the degree of compression of the cerebellar brainstem, and the size of the tumor capsule (Fahy et al., 2002; Del Río et al., 2012; Overdevest et al., 2016). Additionally, VS tumor resection is not effective in eliminating the tinnitus symptoms and a considerable number of patients with VS suffer from further development and deterioration of tinnitus symptoms (Fahy et al., 2002; Del Río et al., 2012; Overdevest et al., 2016). New ways to explain the deterioration of tinnitus in patients with VS are needed.

With the development of neuroimaging technology, more and more central nervous system mechanisms related to tinnitus have been discovered. Gentil et al. (2019) reported that cerebral regional homogeneity was significantly reduced in the contralateral primary auditory cortex and increased in ipsilateral supramarginal and angular gyri to the tinnitus side. Moreover, resting-state network activities involving the auditory network, default mode network, attention networks, visual network, etc., were also confirmed to have significant changes in patients with chronic tinnitus (Kok et al., 2022). Inspired by these neuroimage studies of primary tinnitus, we also conducted a functional gradient study for VS tinnitus. On one hand, in the functional gradient alteration in patients with VS, we observed a bunch of vestibuloocular signatures, including changes in *RH_Vis_1*, *LH_Vis_24*, and *RH_DorsAtten_post_24*. Both *RH_Vis_1* and *LH_Vis_24* are directly involved in visual perception, while *RH_DorsAtten_post_24* of the DA network is well known for its role in visuospatial attention (Vossel et al., 2014). It is closely connected with the intraparietal sulcus and frontal eye field regions, which were proven as two of the main four important components of the vestibular cortex (Dickman, 2018). On the other hand, the alteration in the *RH_DorsAtten_post_24* was quite different from those two visual regions: gradient alterations of *RH_Vis_1* and *LH_Vis_24* regions recovered following surgical resection of the tumor, while gradient alterations of *RH_DorsAtten_post_24* continued to develop. In the group comparison based on tinnitus symptoms, the gradient alterations of *RH_DorsAtten_post_24* were also significant. These studies suggest that disruptions of *RH_DorsAtten_post_24* of the vestibular system may be involved in the maintenance of VS tinnitus.

Subjective tinnitus is a pathology involving neuroplastic changes in central auditory structures that take place when the brain is deprived of its normal input by pathology in the cochlea. There are currently a variety of neural models to explore the occurrence of tinnitus, including the Hyperactivity model, the Tonotopic Reorganization model, the Central Gain model, and the Neural Synchrony model (Henry et al., 2014). The histopathologies or cellular changes that presumably give rise to subjective tinnitus can exist anywhere between the cochlea and auditory cortex, although the majority of cases are triggered by or associated with cochlear-related damage (Rodriguez-Casero et al., 2005). However, long-term maintenance of tinnitus is likely a function of a complex network of structures involving both central auditory and nonauditory systems. Multiple studies demonstrated that bilateral auditory nerve sectioning did not always eliminate tinnitus (Fisch, 1970; Pulec, 1984; Bauer, 2004). Immunolabeling of increased activity was widely found in the brain regions associated with stress such as locus coeruleus, periaqueductal gray, and lateral parabrachial nucleus in animals with salicylate-induced tinnitus (Wallhäusser-Franke, 1997). Over-activity in these areas is closely related to the intensity and duration of sound-induced tinnitus (Chen et al., 2012). The vestibulo-ocular network that arises from the vestibular nuclei is also involved in subjective tinnitus generation (Dickman, 2018; Hu et al., 2021). It was not only found that the disorders of the vestibular nervous system can directly lead to tinnitus (Gavalas et al., 2001), and sometimes tinnitus is the only symptom of vestibular problems (Ila et al., 2019). Therefore, to further alleviating tinnitus symptoms of patients with VS may require us to further intervene in the vestibulo-ocular network.

Several methodological considerations should be contemplated when interpreting these results. This study only included the clinical and imaging data within limited postoperative follow-up. Further longitudinal studies with a larger sample size are required to replicate our findings. Since the cochleovestibular nerve is a mixed nerve, establishing a patient cohort of cochlear nerve-sparing resection makes sense to exclude the effects of different nerve sources. The transcriptome expression data were obtained from healthy participants and were not matched in age and gender. The results from these data might be biased by these variations.

## 5. Conclusion

Functional plasticity of the central nervous system was altered in patients with VS. The functional gradient alteration in the postcentral gyrus region is closely related to tinnitus symptoms in patients with VS. Further understanding of its role in the tinnitus mechanism can not only be used for the prediction of clinical prognosis but also as a potential therapeutic target for these subjective tinnitus.

## Data availability statement

The original contributions presented in this study are included in the article/Supplementary material, further inquiries can be directed to the corresponding author.

## Ethics statement

The studies involving human participants were reviewed and approved by the ethical standards of Ethics Committee in Chinese PLA General Hospital. The patients/participants provided their written informed consent to participate in this study.

## Author contributions

JL: conceptualization, methodology, software, validation, formal analysis, writing—original draft, and visualization. NY: conceptualization, methodology, software, validation, formal analysis, and writing—review and editing. XiaL: methodology, validation, resources, data curation, and writing—review and editing. JiayH, HL, and JianH: investigation and writing—review and editing. JZ: resources, data curation, and writing—review and editing. XinL: supervision, project administration, funding acquisition, and writing—review and editing. All authors contributed to the article and approved the submitted version.

## References

[B1] Andersson G. Kinnefors A. Ekvall L. Rask-Andersen H. (1997). Tinnitus and translabyrinthine acoustic neuroma surgery. *Audiol. Neurootol.* 2 403–409. 10.1159/000259265 9390844

[B2] Badre D. D’Esposito M. (2009). Is the rostro-caudal axis of the frontal lobe hierarchical? *Nat. Rev. Neurosci.* 10 659–669. 10.1038/nrn2667 19672274 PMC3258028

[B3] Baguley D. M. Humphriss R. L. Axon P. R. Moffat D. A. (2006). The clinical characteristics of tinnitus in patients with vestibular schwannoma. *Skull Base* 16 49–58. 17077869 10.1055/s-2005-926216 PMC1502033

[B4] Bauer C. A. (2004). Mechanisms of tinnitus generation. *Curr. Opin. Otolaryngol. Head Neck Surg.* 12 413–417. 10.1097/01.moo.0000134443.29853.09 15377954

[B5] Bayrak Ş Khalil A. A. Villringer K. Fiebach J. B. Villringer A. Margulies D. S. (2019). The impact of ischemic stroke on connectivity gradients. *Neuroimage Clin.* 24:101947. 10.1016/j.nicl.2019.101947 31376644 PMC6676042

[B6] Buckner R. L. Krienen F. M. Castellanos A. Diaz J. C. Yeo B. T. (2011). The organization of the human cerebellum estimated by intrinsic functional connectivity. *J. Neurophysiol.* 106 2322–2345. 10.1152/jn.00339.2011 21795627 PMC3214121

[B7] Chen G. D. Manohar S. Salvi R. (2012). Amygdala hyperactivity and tonotopic shift after salicylate exposure. *Brain Res.* 1485 63–76. 10.1016/j.brainres.2012.03.016 22464181 PMC5319430

[B8] Choi E. Y. Yeo B. T. Buckner R. L. (2012). The organization of the human striatum estimated by intrinsic functional connectivity. *J. Neurophysiol.* 108 2242–2263. 10.1152/jn.00270.2012 22832566 PMC3545026

[B9] Chovanec M. Zvěřina E. Profant O. Balogová Z. Kluh J. Syka J. (2015). Does attempt at hearing preservation microsurgery of vestibular schwannoma affect postoperative tinnitus? *Biomed. Res. Int.* 2015:783169. 10.1155/2015/783169 25654125 PMC4309247

[B10] Del Río L. Lassaletta L. Díaz-Anadón A. Alfonso C. Roda J. M. Gavilán J. (2012). Tinnitus and quality of life following vestibular schwannoma surgery. *B-ENT* 8 167–171. 23113378

[B11] Dickman J. D. (2018). “The vestibular system,” in *Fundamental neuroscience for basic and clinical applications*, eds Haines D. E. Mihailoff G. A. (Amsterdam: Elsevier), 320–333.e1. 10.1016/B978-0-323-39632-5.00022-0

[B12] Fahy C. Nikolopoulos T. P. O’Donoghue G. M. (2002). Acoustic neuroma surgery and tinnitus. *Eur. Arch. Otorhinolaryngol.* 259 299–301. 10.1007/s00405-002-0473-y 12115076

[B13] Fisch U. (1970). Transtemporal surgery of the internal auditory canal. Report of 92 cases, technique, indications and results. *Adv. Otorhinolaryngol.* 17 203–240. 10.1159/000385386 5420552

[B14] Gavalas G. J. Passou E. M. Vathilakis J. M. (2001). Tinnitus of vestibular origin. *Scand. Audiol. Suppl.* 52 185–186. 10.1080/010503901300007470 11318463

[B15] Gentil A. Deverdun J. Menjot de Champfleur N. Puel J. L. Le Bars E. Venail F. (2019). Alterations in regional homogeneity in patients with unilateral chronic tinnitus. *Trends Hear.* 23 2331216519830237. 10.1177/2331216519830237 30995887 PMC6475853

[B16] Henry J. A. Roberts L. E. Caspary D. M. Theodoroff S. M. Salvi R. J. (2014). Underlying mechanisms of tinnitus: Review and clinical implications. *J. Am. Acad. Audiol.* 25 5–22; quiz 126. 10.3766/jaaa.25.1.2 24622858 PMC5063499

[B17] Hong S. J. Vos de Wael R. Bethlehem R. A. I. Lariviere S. Paquola C. Valk S. L. (2019). Atypical functional connectome hierarchy in autism. *Nat. Commun.* 10 1022. 10.1038/s41467-019-08944-1 30833582 PMC6399265

[B18] Hong S. J. Xu T. Nikolaidis A. Smallwood J. Margulies D. S. Bernhardt B. (2020). Toward a connectivity gradient-based framework for reproducible biomarker discovery. *Neuroimage* 223:117322. 10.1016/j.neuroimage.2020.117322 32882388

[B19] Horn A. Kühn A. A. (2015). Lead-DBS: A toolbox for deep brain stimulation electrode localizations and visualizations. *Neuroimage* 107 127–135. 10.1016/j.neuroimage.2014.12.002 25498389

[B20] Hu J. Cui J. Xu J. J. Yin X. Wu Y. Qi J. (2021). The neural mechanisms of tinnitus: A perspective from functional magnetic resonance imaging. *Front. Neurosci.* 15:621145. 10.3389/fnins.2021.621145 33642982 PMC7905063

[B21] Huntenburg J. M. Bazin P. L. Margulies D. S. (2018). Large-scale gradients in human cortical organization. *Trends Cogn. Sci.* 22 21–31. 10.1016/j.tics.2017.11.002 29203085

[B22] Ila K. Soylemez E. Yilmaz N. Kayis S. A. Eshraghi A. A. (2019). Vestibular functions in patients with tinnitus only. *Acta Otolaryngol.* 139 162–166. 30734617 10.1080/00016489.2018.1548778

[B23] Kok T. E. Domingo D. Hassan J. Vuong A. Hordacre B. Clark C. (2022). Resting-state networks in tinnitus: A scoping review. *Clin. Neuroradiol.* 32 903–922. 10.1007/s00062-022-01170-1 35556148 PMC9744700

[B24] Liao Y. Wang J. Jaehnig E. J. Shi Z. Zhang B. (2019). WebGestalt 2019: Gene set analysis toolkit with revamped UIs and APIs. *Nucleic Acids Res.* 47 W199–W205. 10.1093/nar/gkz401 31114916 PMC6602449

[B25] Margulies D. S. Ghosh S. S. Goulas A. Falkiewicz M. Huntenburg J. M. Langs G. (2016). Situating the default-mode network along a principal gradient of macroscale cortical organization. *Proc. Natl. Acad. Sci. U.S.A.* 113 12574–12579. 10.1073/pnas.1608282113 27791099 PMC5098630

[B26] Markello R. D. Arnatkeviciute A. Poline J. B. Fulcher B. D. Fornito A. Misic B. (2021). Standardizing workflows in imaging transcriptomics with the abagen toolbox. *Elife* 10:e72129. 10.7554/eLife.72129 34783653 PMC8660024

[B27] Meng Y. Yang S. Chen H. Li J. Xu Q. Zhang Q. (2021). Systematically disrupted functional gradient of the cortical connectome in generalized epilepsy: Initial discovery and independent sample replication. *Neuroimage* 230:117831. 10.1016/j.neuroimage.2021.117831 33549757

[B28] Mishkin M. Ungerleider L. G. (1982). Contribution of striate inputs to the visuospatial functions of parieto-preoccipital cortex in monkeys. *Behav. Brain Res.* 6 57–77. 10.1016/0166-4328(82)90081-X 7126325

[B29] Newman C. W. Jacobson G. P. Spitzer J. B. (1996). Development of the tinnitus handicap inventory. *Arch. Otolaryngol. Head Neck Surg.* 122 143–148. 10.1001/archotol.1996.01890140029007 8630207

[B30] Overdevest J. B. Pross S. E. Cheung S. W. (2016). Tinnitus following treatment for sporadic acoustic neuroma. *Laryngoscope* 126 1639–1643. 10.1002/lary.25672 26403598

[B31] Pulec J. L. (1984). Tinnitus: Surgical therapy. *Am. J. Otol.* 5 479–480. 6517136

[B32] Raj-Koziak D. Gos E. Swierniak W. Rajchel J. J. Karpiesz L. Niedzialek I. (2018). Visual analogue scales as a tool for initial assessment of tinnitus severity: Psychometric evaluation in a clinical population. *Audiol. Neurootol.* 23 229–237. 10.1159/000494021 30439712 PMC6381860

[B33] Rodriguez-Casero M. V. Mandelstam S. Kornberg A. J. Berkowitz R. G. (2005). Acute tinnitus and hearing loss as the initial symptom of multiple sclerosis in a child. *Int. J. Pediatr. Otorhinolaryngol.* 69 123–126. 10.1016/j.ijporl.2004.08.006 15627460

[B34] Schaefer A. Kong R. Gordon E. M. Laumann T. O. Zuo X. N. Holmes A. J. (2018). Local-global parcellation of the human cerebral cortex from intrinsic functional connectivity MRI. *Cereb. Cortex* 28 3095–3114. 10.1093/cercor/bhx179 28981612 PMC6095216

[B35] Stankovic K. M. Mrugala M. M. Martuza R. L. Silver M. Betensky R. A. Nadol J. B. Jr. (2009). Genetic determinants of hearing loss associated with vestibular schwannomas. *Otol. Neurotol.* 30 661–667. 10.1097/MAO.0b013e3181a66ece 19546833

[B36] Stipkovits E. M. van Dijk J. E. Graamans K. (1998). Profile of hearing in patients with unilateral acoustic neuromas: The importance of the contralateral ear. *Am. J. Otol.* 19 834–839. 9831164

[B37] van Wijk B. C. Stam C. J. Daffertshofer A. (2010). Comparing brain networks of different size and connectivity density using graph theory. *PLoS One* 5:e13701. 10.1371/journal.pone.0013701 21060892 PMC2965659

[B38] Vos de Wael R. Benkarim O. Paquola C. Lariviere S. Royer J. Tavakol S. (2020). BrainSpace: A toolbox for the analysis of macroscale gradients in neuroimaging and connectomics datasets. *Commun. Biol.* 3:103. 10.1038/s42003-020-0794-7 32139786 PMC7058611

[B39] Vossel S. Geng J. J. Fink G. R. (2014). Dorsal and ventral attention systems: Distinct neural circuits but collaborative roles. *Neuroscientist* 20 150–159. 10.1177/1073858413494269 23835449 PMC4107817

[B40] Wallhäusser-Franke E. (1997). Salicylate evokes c-fos expression in the brain stem: Implications for tinnitus. *Neuroreport* 8 725–728. 10.1097/00001756-199702100-00029 9106755

[B41] Wang J. Wang X. Xia M. Liao X. Evans A. He Y. (2015). GRETNA: A graph theoretical network analysis toolbox for imaging connectomics. *Front. Hum. Neurosci.* 9:386. 10.3389/fnhum.2015.00386 26175682 PMC4485071

[B42] Wang J. Zhou Y. Ding J. Xiao J. (2021). Functional gradient alteration in individuals with cognitive vulnerability to depression. *J. Psychiatr. Res.* 144 338–344. 10.1016/j.jpsychires.2021.10.024 34735837

[B43] Wang X. Zeng R. Zhuang H. Sun Q. Yang Z. Sun C. (2020). Chinese validation and clinical application of the tinnitus functional index. *Health Qual. Life Outcomes* 18:272. 10.1186/s12955-020-01514-w 32762753 PMC7409716

[B44] Yeo B. T. Krienen F. M. Sepulcre J. Sabuncu M. R. Lashkari D. Hollinshead M. (2011). The organization of the human cerebral cortex estimated by intrinsic functional connectivity. *J. Neurophysiol.* 106 1125–1165. 10.1152/jn.00338.2011 21653723 PMC3174820

