# Corrigendum: Atypical functional hierarchy contributed to the tinnitus symptoms in patients with vestibular schwannoma

**DOI:** 10.3389/fnins.2023.1251234

**Published:** 2023-07-14

**Authors:** Jiaji Lin, Na You, Xiaolong Li, Jiayu Huang, Haoxuan Lu, Jianxing Hu, Jun Zhang, Xin Lou

**Affiliations:** ^1^Department of Radiology, Chinese People's Liberation Army General Hospital/Medical School of Chinese People's Liberation Army, Beijing, China; ^2^Department of Neurosurgery, Chinese People's Liberation Army General Hospital/Medical School of Chinese People's Liberation Army, Beijing, China

**Keywords:** functional gradient, tinnitus, tinnitus handicap inventory, visual analog scale, vestibular schwannoma

In the published article, there was an error in the author list, and author “Jun Zhang” was erroneously excluded as a co-corresponding author. The corrected author list appears below.

“Jiaji Lin^1^^†^, Na You^2^^†^, Xiaolong Li^1^^†^, Jiayu Huang^1^^†^, Haoxuan Lu^1^, Jianxing Hu^1^, Jun Zhang^2*^ and Xin Lou^1*^”

The authors apologize for this error and state that this does not change the scientific conclusions of the article in any way. The original article has been updated.

