# Supplementary material for: Atypical functional hierarchy contributed to the tinnitus symptoms in patients with vestibular schwannoma
Source: Front Neurosci. 2023 Feb 17;17:1084270. doi: 10.3389/fnins.2023.1084270 (PMC9982843; doi:10.3389/fnins.2023.1084270)
Supplement: Supplementary file 1 [file Data_Sheet_1.pdf]

Supplementary Materials for

Atypical functional hierarchy contributed to the tinnitus symptoms in patients with vestibular schwannoma

This PDF file includes:

Figure. S1

Tables. S1 to S9

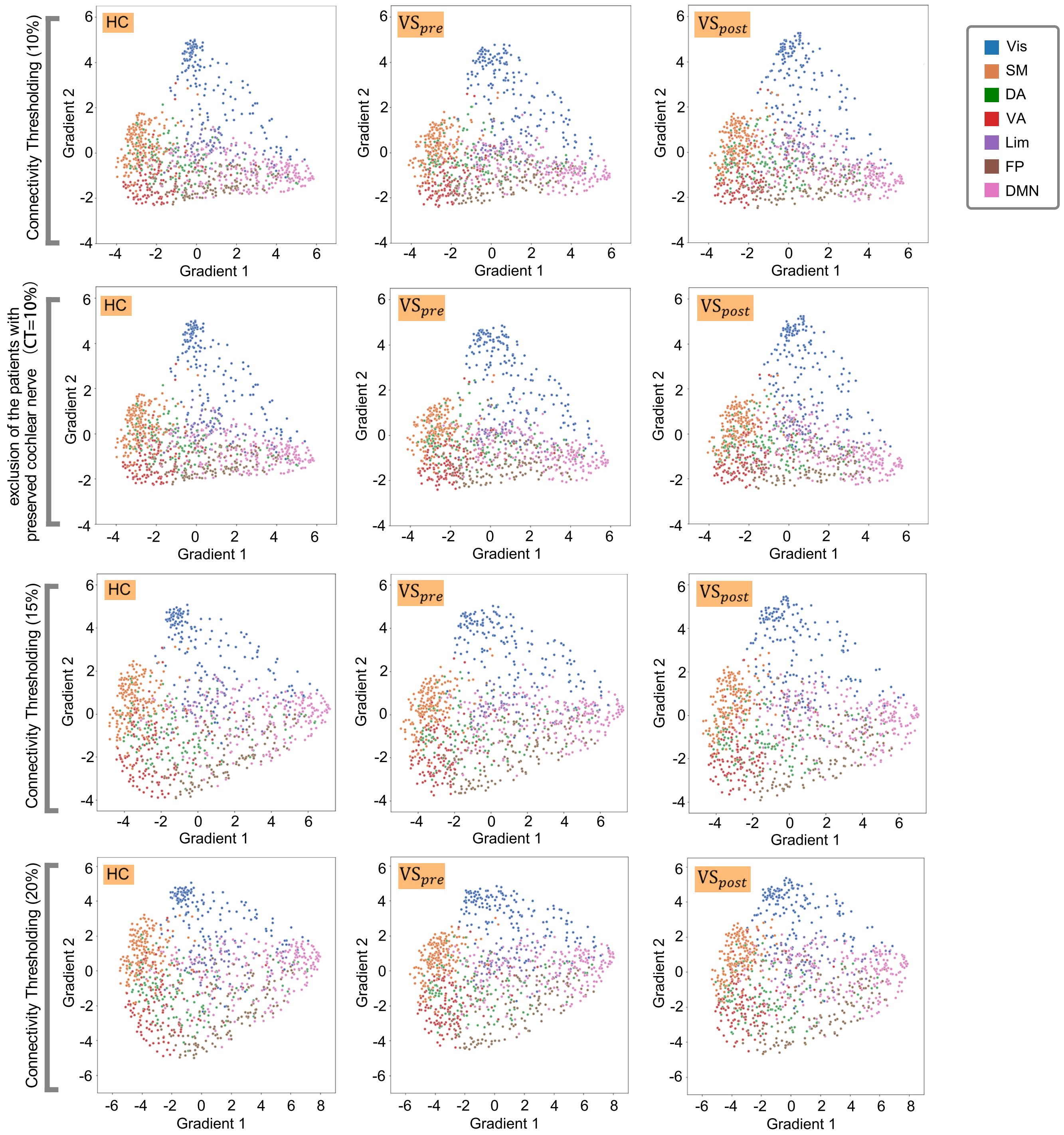

Connectivity Thresholding (10%)
exclusion of the patients with preserved cochlear nerve (CT=10%)
Connectivity Thresholding (15%)
Connectivity Thresholding (20%)
HC
VSpre
VSpost
Gradient 1
Gradient 2
Vis
SM
DA
VA
Lim
FP
DMN

Supplementary Figure 1. Perturbed global gradients in VS. Global gradients alterations were virtually identical when controlling for connectivity matrix thresholding (10–20%) or exclusion of the patients with preserved cochlear nerve.

Table S1 Atlas annotation

ROI Label	ROI Name	Yeo community	Atlas source
1	7Networks_LH_Vis_1	Vis	Schaefer
2	7Networks_LH_Vis_2	Vis	Schaefer
3	7Networks_LH_Vis_3	Vis	Schaefer
4	7Networks_LH_Vis_4	Vis	Schaefer
5	7Networks_LH_Vis_5	Vis	Schaefer
6	7Networks_LH_Vis_6	Vis	Schaefer
7	7Networks_LH_Vis_7	Vis	Schaefer
8	7Networks_LH_Vis_8	Vis	Schaefer
9	7Networks_LH_Vis_9	Vis	Schaefer
10	7Networks_LH_Vis_10	Vis	Schaefer
11	7Networks_LH_Vis_11	Vis	Schaefer
12	7Networks_LH_Vis_12	Vis	Schaefer
13	7Networks_LH_Vis_13	Vis	Schaefer
14	7Networks_LH_Vis_14	Vis	Schaefer
15	7Networks_LH_Vis_15	Vis	Schaefer
16	7Networks_LH_Vis_16	Vis	Schaefer
17	7Networks_LH_Vis_17	Vis	Schaefer
18	7Networks_LH_Vis_18	Vis	Schaefer
19	7Networks_LH_Vis_19	Vis	Schaefer
20	7Networks_LH_Vis_20	Vis	Schaefer
21	7Networks_LH_Vis_21	Vis	Schaefer
22	7Networks_LH_Vis_22	Vis	Schaefer
23	7Networks_LH_Vis_23	Vis	Schaefer
24	7Networks_LH_Vis_24	Vis	Schaefer
25	7Networks_LH_Vis_25	Vis	Schaefer
26	7Networks_LH_Vis_26	Vis	Schaefer
27	7Networks_LH_Vis_27	Vis	Schaefer
28	7Networks_LH_Vis_28	Vis	Schaefer
29	7Networks_LH_Vis_29	Vis	Schaefer
30	7Networks_LH_Vis_30	Vis	Schaefer
31	7Networks_LH_Vis_31	Vis	Schaefer
32	7Networks_LH_Vis_32	Vis	Schaefer
33	7Networks_LH_Vis_33	Vis	Schaefer
34	7Networks_LH_Vis_34	Vis	Schaefer
35	7Networks_LH_Vis_35	Vis	Schaefer
36	7Networks_LH_Vis_36	Vis	Schaefer
37	7Networks_LH_Vis_37	Vis	Schaefer
38	7Networks_LH_Vis_38	Vis	Schaefer
39	7Networks_LH_Vis_39	Vis	Schaefer
40	7Networks_LH_Vis_40	Vis	Schaefer
41	7Networks_LH_Vis_41	Vis	Schaefer
42	7Networks_LH_Vis_42	Vis	Schaefer

43	7Networks_LH_Vis_43	Vis	Schaefer
44	7Networks_LH_Vis_44	Vis	Schaefer
45	7Networks_LH_Vis_45	Vis	Schaefer
46	7Networks_LH_Vis_46	Vis	Schaefer
47	7Networks_LH_Vis_47	Vis	Schaefer
48	7Networks_LH_Vis_48	Vis	Schaefer
49	7Networks_LH_Vis_49	Vis	Schaefer
50	7Networks_LH_Vis_50	Vis	Schaefer
51	7Networks_LH_Vis_51	Vis	Schaefer
52	7Networks_LH_Vis_52	Vis	Schaefer
53	7Networks_LH_Vis_53	Vis	Schaefer
54	7Networks_LH_Vis_54	Vis	Schaefer
55	7Networks_LH_Vis_55	Vis	Schaefer
56	7Networks_LH_Vis_56	Vis	Schaefer
57	7Networks_LH_Vis_57	Vis	Schaefer
58	7Networks_LH_Vis_58	Vis	Schaefer
59	7Networks_LH_Vis_59	Vis	Schaefer
60	7Networks_LH_Vis_60	Vis	Schaefer
61	7Networks_LH_Vis_61	Vis	Schaefer
62	7Networks_LH_Vis_62	Vis	Schaefer
63	7Networks_LH_Vis_63	Vis	Schaefer
64	7Networks_LH_Vis_64	Vis	Schaefer
65	7Networks_LH_Vis_65	Vis	Schaefer
66	7Networks_LH_Vis_66	Vis	Schaefer
67	7Networks_LH_Vis_67	Vis	Schaefer
68	7Networks_LH_Vis_68	Vis	Schaefer
69	7Networks_LH_Vis_69	Vis	Schaefer
70	7Networks_LH_Vis_70	Vis	Schaefer
71	7Networks_LH_Vis_71	Vis	Schaefer
72	7Networks_LH_Vis_72	Vis	Schaefer
73	7Networks_LH_Vis_73	Vis	Schaefer
74	7Networks_LH_Vis_74	Vis	Schaefer
75	7Networks_LH_Vis_75	Vis	Schaefer
76	7Networks_LH_Vis_76	Vis	Schaefer
77	7Networks_LH_Vis_77	Vis	Schaefer
78	7Networks_LH_Vis_78	Vis	Schaefer
79	7Networks_LH_Vis_79	Vis	Schaefer
80	7Networks_LH_Vis_80	Vis	Schaefer
81	7Networks_LH_Vis_81	Vis	Schaefer
82	7Networks_LH_SomMot_1	SomMot	Schaefer
83	7Networks_LH_SomMot_2	SomMot	Schaefer
84	7Networks_LH_SomMot_3	SomMot	Schaefer
85	7Networks_LH_SomMot_4	SomMot	Schaefer
86	7Networks_LH_SomMot_5	SomMot	Schaefer

87	7Networks_LH_SomMot_6	SomMot	Schaefer
88	7Networks_LH_SomMot_7	SomMot	Schaefer
89	7Networks_LH_SomMot_8	SomMot	Schaefer
90	7Networks_LH_SomMot_9	SomMot	Schaefer
91	7Networks_LH_SomMot_10	SomMot	Schaefer
92	7Networks_LH_SomMot_11	SomMot	Schaefer
93	7Networks_LH_SomMot_12	SomMot	Schaefer
94	7Networks_LH_SomMot_13	SomMot	Schaefer
95	7Networks_LH_SomMot_14	SomMot	Schaefer
96	7Networks_LH_SomMot_15	SomMot	Schaefer
97	7Networks_LH_SomMot_16	SomMot	Schaefer
98	7Networks_LH_SomMot_17	SomMot	Schaefer
99	7Networks_LH_SomMot_18	SomMot	Schaefer
100	7Networks_LH_SomMot_19	SomMot	Schaefer
101	7Networks_LH_SomMot_20	SomMot	Schaefer
102	7Networks_LH_SomMot_21	SomMot	Schaefer
103	7Networks_LH_SomMot_22	SomMot	Schaefer
104	7Networks_LH_SomMot_23	SomMot	Schaefer
105	7Networks_LH_SomMot_24	SomMot	Schaefer
106	7Networks_LH_SomMot_25	SomMot	Schaefer
107	7Networks_LH_SomMot_26	SomMot	Schaefer
108	7Networks_LH_SomMot_27	SomMot	Schaefer
109	7Networks_LH_SomMot_28	SomMot	Schaefer
110	7Networks_LH_SomMot_29	SomMot	Schaefer
111	7Networks_LH_SomMot_30	SomMot	Schaefer
112	7Networks_LH_SomMot_31	SomMot	Schaefer
113	7Networks_LH_SomMot_32	SomMot	Schaefer
114	7Networks_LH_SomMot_33	SomMot	Schaefer
115	7Networks_LH_SomMot_34	SomMot	Schaefer
116	7Networks_LH_SomMot_35	SomMot	Schaefer
117	7Networks_LH_SomMot_36	SomMot	Schaefer
118	7Networks_LH_SomMot_37	SomMot	Schaefer
119	7Networks_LH_SomMot_38	SomMot	Schaefer
120	7Networks_LH_SomMot_39	SomMot	Schaefer
121	7Networks_LH_SomMot_40	SomMot	Schaefer
122	7Networks_LH_SomMot_41	SomMot	Schaefer
123	7Networks_LH_SomMot_42	SomMot	Schaefer
124	7Networks_LH_SomMot_43	SomMot	Schaefer
125	7Networks_LH_SomMot_44	SomMot	Schaefer
126	7Networks_LH_SomMot_45	SomMot	Schaefer
127	7Networks_LH_SomMot_46	SomMot	Schaefer
128	7Networks_LH_SomMot_47	SomMot	Schaefer
129	7Networks_LH_SomMot_48	SomMot	Schaefer
130	7Networks_LH_SomMot_49	SomMot	Schaefer

131	7Networks_LH_SomMot_50	SomMot	Schaefer
132	7Networks_LH_SomMot_51	SomMot	Schaefer
133	7Networks_LH_SomMot_52	SomMot	Schaefer
134	7Networks_LH_SomMot_53	SomMot	Schaefer
135	7Networks_LH_SomMot_54	SomMot	Schaefer
136	7Networks_LH_SomMot_55	SomMot	Schaefer
137	7Networks_LH_SomMot_56	SomMot	Schaefer
138	7Networks_LH_SomMot_57	SomMot	Schaefer
139	7Networks_LH_SomMot_58	SomMot	Schaefer
140	7Networks_LH_SomMot_59	SomMot	Schaefer
141	7Networks_LH_SomMot_60	SomMot	Schaefer
142	7Networks_LH_SomMot_61	SomMot	Schaefer
143	7Networks_LH_SomMot_62	SomMot	Schaefer
144	7Networks_LH_SomMot_63	SomMot	Schaefer
145	7Networks_LH_SomMot_64	SomMot	Schaefer
146	7Networks_LH_SomMot_65	SomMot	Schaefer
147	7Networks_LH_SomMot_66	SomMot	Schaefer
148	7Networks_LH_SomMot_67	SomMot	Schaefer
149	7Networks_LH_SomMot_68	SomMot	Schaefer
150	7Networks_LH_SomMot_69	SomMot	Schaefer
151	7Networks_LH_SomMot_70	SomMot	Schaefer
152	7Networks_LH_SomMot_71	SomMot	Schaefer
153	7Networks_LH_SomMot_72	SomMot	Schaefer
154	7Networks_LH_SomMot_73	SomMot	Schaefer
155	7Networks_LH_SomMot_74	SomMot	Schaefer
156	7Networks_LH_SomMot_75	SomMot	Schaefer
157	7Networks_LH_SomMot_76	SomMot	Schaefer
158	7Networks_LH_SomMot_77	SomMot	Schaefer
159	7Networks_LH_SomMot_78	SomMot	Schaefer
160	7Networks_LH_SomMot_79	SomMot	Schaefer
161	7Networks_LH_SomMot_80	SomMot	Schaefer
162	7Networks_LH_SomMot_81	SomMot	Schaefer
163	7Networks_LH_SomMot_82	SomMot	Schaefer
164	7Networks_LH_SomMot_83	SomMot	Schaefer
165	7Networks_LH_SomMot_84	SomMot	Schaefer
166	7Networks_LH_SomMot_85	SomMot	Schaefer
167	7Networks_LH_SomMot_86	SomMot	Schaefer
168	7Networks_LH_SomMot_87	SomMot	Schaefer
169	7Networks_LH_SomMot_88	SomMot	Schaefer
170	7Networks_LH_SomMot_89	SomMot	Schaefer
171	7Networks_LH_SomMot_90	SomMot	Schaefer
172	7Networks_LH_SomMot_91	SomMot	Schaefer
173	7Networks_LH_DorsAttn_Post_1	SalVentAt	Schaefer
174	7Networks_LH_DorsAttn_Post_2	SalVentAt	Schaefer

175	7Networks_LH_DorsAttn_Post_3	SalVentAt	Schaefer
176	7Networks_LH_DorsAttn_Post_4	SalVentAt	Schaefer
177	7Networks_LH_DorsAttn_Post_5	SalVentAt	Schaefer
178	7Networks_LH_DorsAttn_Post_6	SalVentAt	Schaefer
179	7Networks_LH_DorsAttn_Post_7	SalVentAt	Schaefer
180	7Networks_LH_DorsAttn_Post_8	SalVentAt	Schaefer
181	7Networks_LH_DorsAttn_Post_9	SalVentAt	Schaefer
182	7Networks_LH_DorsAttn_Post_10	SalVentAt	Schaefer
183	7Networks_LH_DorsAttn_Post_11	SalVentAt	Schaefer
184	7Networks_LH_DorsAttn_Post_12	SalVentAt	Schaefer
185	7Networks_LH_DorsAttn_Post_13	SalVentAt	Schaefer
186	7Networks_LH_DorsAttn_Post_14	SalVentAt	Schaefer
187	7Networks_LH_DorsAttn_Post_15	SalVentAt	Schaefer
188	7Networks_LH_DorsAttn_Post_16	SalVentAt	Schaefer
189	7Networks_LH_DorsAttn_Post_17	SalVentAt	Schaefer
190	7Networks_LH_DorsAttn_Post_18	SalVentAt	Schaefer
191	7Networks_LH_DorsAttn_Post_19	SalVentAt	Schaefer
192	7Networks_LH_DorsAttn_Post_20	SalVentAt	Schaefer
193	7Networks_LH_DorsAttn_Post_21	SalVentAt	Schaefer
194	7Networks_LH_DorsAttn_Post_22	SalVentAt	Schaefer
195	7Networks_LH_DorsAttn_Post_23	SalVentAt	Schaefer
196	7Networks_LH_DorsAttn_Post_24	SalVentAt	Schaefer
197	7Networks_LH_DorsAttn_Post_25	SalVentAt	Schaefer
198	7Networks_LH_DorsAttn_Post_26	SalVentAt	Schaefer
199	7Networks_LH_DorsAttn_Post_27	SalVentAt	Schaefer
200	7Networks_LH_DorsAttn_Post_28	SalVentAt	Schaefer
201	7Networks_LH_DorsAttn_Post_29	SalVentAt	Schaefer
202	7Networks_LH_DorsAttn_Post_30	SalVentAt	Schaefer
203	7Networks_LH_DorsAttn_Post_31	SalVentAt	Schaefer
204	7Networks_LH_DorsAttn_Post_32	SalVentAt	Schaefer
205	7Networks_LH_DorsAttn_Post_33	SalVentAt	Schaefer
206	7Networks_LH_DorsAttn_Post_34	SalVentAt	Schaefer
207	7Networks_LH_DorsAttn_Post_35	SalVentAt	Schaefer
208	7Networks_LH_DorsAttn_Post_36	SalVentAt	Schaefer
209	7Networks_LH_DorsAttn_Post_37	SalVentAt	Schaefer
210	7Networks_LH_DorsAttn_Post_38	SalVentAt	Schaefer
211	7Networks_LH_DorsAttn_Post_39	SalVentAt	Schaefer
212	7Networks_LH_DorsAttn_Post_40	SalVentAt	Schaefer
213	7Networks_LH_DorsAttn_Post_41	SalVentAt	Schaefer
214	7Networks_LH_DorsAttn_Post_42	SalVentAt	Schaefer
215	7Networks_LH_DorsAttn_Post_43	SalVentAt	Schaefer
216	7Networks_LH_DorsAttn_Post_44	SalVentAt	Schaefer
217	7Networks_LH_DorsAttn_Post_45	SalVentAt	Schaefer
218	7Networks_LH_DorsAttn_Post_46	SalVentAt	Schaefer

219	7Networks_LH_DorsAttn_Post_47	SalVentAt	Schaefer
220	7Networks_LH_DorsAttn_Post_48	SalVentAt	Schaefer
221	7Networks_LH_DorsAttn_Post_49	SalVentAt	Schaefer
222	7Networks_LH_DorsAttn_Post_50	SalVentAt	Schaefer
223	7Networks_LH_DorsAttn_FEF_1	SalVentAt	Schaefer
224	7Networks_LH_DorsAttn_FEF_2	SalVentAt	Schaefer
225	7Networks_LH_DorsAttn_FEF_3	SalVentAt	Schaefer
226	7Networks_LH_DorsAttn_FEF_4	SalVentAt	Schaefer
227	7Networks_LH_DorsAttn_FEF_5	SalVentAt	Schaefer
228	7Networks_LH_DorsAttn_FEF_6	SalVentAt	Schaefer
229	7Networks_LH_DorsAttn_FEF_7	SalVentAt	Schaefer
230	7Networks_LH_DorsAttn_PrCv_1	SalVentAt	Schaefer
231	7Networks_LH_DorsAttn_PrCv_2	SalVentAt	Schaefer
232	7Networks_LH_DorsAttn_PrCv_3	SalVentAt	Schaefer
233	7Networks_LH_DorsAttn_PrCv_4	SalVentAt	Schaefer
234	7Networks_LH_SalVentAttn_ParOper_1	DorsAttn	Schaefer
235	7Networks_LH_SalVentAttn_ParOper_2	DorsAttn	Schaefer
236	7Networks_LH_SalVentAttn_ParOper_3	DorsAttn	Schaefer
237	7Networks_LH_SalVentAttn_ParOper_4	DorsAttn	Schaefer
238	7Networks_LH_SalVentAttn_ParOper_5	DorsAttn	Schaefer
239	7Networks_LH_SalVentAttn_ParOper_6	DorsAttn	Schaefer
240	7Networks_LH_SalVentAttn_ParOper_7	DorsAttn	Schaefer
241	7Networks_LH_SalVentAttn_ParOper_8	DorsAttn	Schaefer
242	7Networks_LH_SalVentAttn_ParOper_9	DorsAttn	Schaefer
243	7Networks_LH_SalVentAttn_TempOcc_1	DorsAttn	Schaefer
244	7Networks_LH_SalVentAttn_TempOcc_2	DorsAttn	Schaefer
245	7Networks_LH_SalVentAttn_FrOperIns_1	DorsAttn	Schaefer
246	7Networks_LH_SalVentAttn_FrOperIns_2	DorsAttn	Schaefer
247	7Networks_LH_SalVentAttn_FrOperIns_3	DorsAttn	Schaefer
248	7Networks_LH_SalVentAttn_FrOperIns_4	DorsAttn	Schaefer
249	7Networks_LH_SalVentAttn_FrOperIns_5	DorsAttn	Schaefer
250	7Networks_LH_SalVentAttn_FrOperIns_6	DorsAttn	Schaefer
251	7Networks_LH_SalVentAttn_FrOperIns_7	DorsAttn	Schaefer
252	7Networks_LH_SalVentAttn_FrOperIns_8	DorsAttn	Schaefer
253	7Networks_LH_SalVentAttn_FrOperIns_9	DorsAttn	Schaefer
254	7Networks_LH_SalVentAttn_FrOperIns_10	DorsAttn	Schaefer
255	7Networks_LH_SalVentAttn_FrOperIns_11	DorsAttn	Schaefer
256	7Networks_LH_SalVentAttn_FrOperIns_12	DorsAttn	Schaefer
257	7Networks_LH_SalVentAttn_FrOperIns_13	DorsAttn	Schaefer
258	7Networks_LH_SalVentAttn_FrOperIns_14	DorsAttn	Schaefer
259	7Networks_LH_SalVentAttn_FrOperIns_15	DorsAttn	Schaefer
260	7Networks_LH_SalVentAttn_FrOperIns_16	DorsAttn	Schaefer
261	7Networks_LH_SalVentAttn_FrOperIns_17	DorsAttn	Schaefer
262	7Networks_LH_SalVentAttn_FrOperIns_18	DorsAttn	Schaefer

263	7Networks_LH_SalVentAttn_FrOperIns_19	DorsAttn	Schaefer
264	7Networks_LH_SalVentAttn_FrOperIns_20	DorsAttn	Schaefer
265	7Networks_LH_SalVentAttn_FrOperIns_21	DorsAttn	Schaefer
266	7Networks_LH_SalVentAttn_FrOperIns_22	DorsAttn	Schaefer
267	7Networks_LH_SalVentAttn_FrOperIns_23	DorsAttn	Schaefer
268	7Networks_LH_SalVentAttn_PFCl_1	DorsAttn	Schaefer
269	7Networks_LH_SalVentAttn_PFCl_2	DorsAttn	Schaefer
270	7Networks_LH_SalVentAttn_Med_1	DorsAttn	Schaefer
271	7Networks_LH_SalVentAttn_Med_2	DorsAttn	Schaefer
272	7Networks_LH_SalVentAttn_Med_3	DorsAttn	Schaefer
273	7Networks_LH_SalVentAttn_Med_4	DorsAttn	Schaefer
274	7Networks_LH_SalVentAttn_Med_5	DorsAttn	Schaefer
275	7Networks_LH_SalVentAttn_Med_6	DorsAttn	Schaefer
276	7Networks_LH_SalVentAttn_Med_7	DorsAttn	Schaefer
277	7Networks_LH_SalVentAttn_Med_8	DorsAttn	Schaefer
278	7Networks_LH_SalVentAttn_Med_9	DorsAttn	Schaefer
279	7Networks_LH_SalVentAttn_Med_10	DorsAttn	Schaefer
280	7Networks_LH_SalVentAttn_Med_11	DorsAttn	Schaefer
281	7Networks_LH_SalVentAttn_Med_12	DorsAttn	Schaefer
282	7Networks_LH_SalVentAttn_Med_13	DorsAttn	Schaefer
283	7Networks_LH_SalVentAttn_Med_14	DorsAttn	Schaefer
284	7Networks_LH_SalVentAttn_Med_15	DorsAttn	Schaefer
285	7Networks_LH_SalVentAttn_Med_16	DorsAttn	Schaefer
286	7Networks_LH_SalVentAttn_Med_17	DorsAttn	Schaefer
287	7Networks_LH_SalVentAttn_Med_18	DorsAttn	Schaefer
288	7Networks_LH_SalVentAttn_Med_19	DorsAttn	Schaefer
289	7Networks_LH_Limbic_OFC_1	Limbic	Schaefer
290	7Networks_LH_Limbic_OFC_2	Limbic	Schaefer
291	7Networks_LH_Limbic_OFC_3	Limbic	Schaefer
292	7Networks_LH_Limbic_OFC_4	Limbic	Schaefer
293	7Networks_LH_Limbic_OFC_5	Limbic	Schaefer
294	7Networks_LH_Limbic_OFC_6	Limbic	Schaefer
295	7Networks_LH_Limbic_OFC_7	Limbic	Schaefer
296	7Networks_LH_Limbic_OFC_8	Limbic	Schaefer
297	7Networks_LH_Limbic_OFC_9	Limbic	Schaefer
298	7Networks_LH_Limbic_OFC_10	Limbic	Schaefer
299	7Networks_LH_Limbic_OFC_11	Limbic	Schaefer
300	7Networks_LH_Limbic_OFC_12	Limbic	Schaefer
301	7Networks_LH_Limbic_OFC_13	Limbic	Schaefer
302	7Networks_LH_Limbic_OFC_14	Limbic	Schaefer
303	7Networks_LH_Limbic_TempPole_1	Limbic	Schaefer
304	7Networks_LH_Limbic_TempPole_2	Limbic	Schaefer
305	7Networks_LH_Limbic_TempPole_3	Limbic	Schaefer
306	7Networks_LH_Limbic_TempPole_4	Limbic	Schaefer

307	7Networks_LH_Limbic_TempPole_5	Limbic	Schaefer
308	7Networks_LH_Limbic_TempPole_6	Limbic	Schaefer
309	7Networks_LH_Limbic_TempPole_7	Limbic	Schaefer
310	7Networks_LH_Limbic_TempPole_8	Limbic	Schaefer
311	7Networks_LH_Limbic_TempPole_9	Limbic	Schaefer
312	7Networks_LH_Limbic_TempPole_10	Limbic	Schaefer
313	7Networks_LH_Limbic_TempPole_11	Limbic	Schaefer
314	7Networks_LH_Limbic_TempPole_12	Limbic	Schaefer
315	7Networks_LH_Limbic_TempPole_13	Limbic	Schaefer
316	7Networks_LH_Limbic_TempPole_14	Limbic	Schaefer
317	7Networks_LH_Limbic_TempPole_15	Limbic	Schaefer
318	7Networks_LH_Cont_Par_1	Cont	Schaefer
319	7Networks_LH_Cont_Par_2	Cont	Schaefer
320	7Networks_LH_Cont_Par_3	Cont	Schaefer
321	7Networks_LH_Cont_Par_4	Cont	Schaefer
322	7Networks_LH_Cont_Par_5	Cont	Schaefer
323	7Networks_LH_Cont_Par_6	Cont	Schaefer
324	7Networks_LH_Cont_Par_7	Cont	Schaefer
325	7Networks_LH_Cont_Par_8	Cont	Schaefer
326	7Networks_LH_Cont_Par_9	Cont	Schaefer
327	7Networks_LH_Cont_Par_10	Cont	Schaefer
328	7Networks_LH_Cont_Par_11	Cont	Schaefer
329	7Networks_LH_Cont_Par_12	Cont	Schaefer
330	7Networks_LH_Cont_Par_13	Cont	Schaefer
331	7Networks_LH_Cont_Par_14	Cont	Schaefer
332	7Networks_LH_Cont_Par_15	Cont	Schaefer
333	7Networks_LH_Cont_Temp_1	Cont	Schaefer
334	7Networks_LH_Cont_Temp_2	Cont	Schaefer
335	7Networks_LH_Cont_Temp_3	Cont	Schaefer
336	7Networks_LH_Cont_Temp_4	Cont	Schaefer
337	7Networks_LH_Cont_PFCd_1	Cont	Schaefer
338	7Networks_LH_Cont_PFCl_1	Cont	Schaefer
339	7Networks_LH_Cont_PFCl_2	Cont	Schaefer
340	7Networks_LH_Cont_PFCl_3	Cont	Schaefer
341	7Networks_LH_Cont_PFCl_4	Cont	Schaefer
342	7Networks_LH_Cont_PFCl_5	Cont	Schaefer
343	7Networks_LH_Cont_PFCl_6	Cont	Schaefer
344	7Networks_LH_Cont_PFCl_7	Cont	Schaefer
345	7Networks_LH_Cont_PFCl_8	Cont	Schaefer
346	7Networks_LH_Cont_PFCl_9	Cont	Schaefer
347	7Networks_LH_Cont_PFCl_10	Cont	Schaefer
348	7Networks_LH_Cont_PFCl_11	Cont	Schaefer
349	7Networks_LH_Cont_PFCl_12	Cont	Schaefer
350	7Networks_LH_Cont_PFCl_13	Cont	Schaefer

351	7Networks_LH_Cont_PFCl_14	Cont	Schaefer
352	7Networks_LH_Cont_PFCl_15	Cont	Schaefer
353	7Networks_LH_Cont_PFCl_16	Cont	Schaefer
354	7Networks_LH_Cont_PFCl_17	Cont	Schaefer
355	7Networks_LH_Cont_PFCl_18	Cont	Schaefer
356	7Networks_LH_Cont_PFCl_19	Cont	Schaefer
357	7Networks_LH_Cont_PFCl_20	Cont	Schaefer
358	7Networks_LH_Cont_OFC_1	Cont	Schaefer
359	7Networks_LH_Cont_PFCv_1	Cont	Schaefer
360	7Networks_LH_Cont_PFCv_2	Cont	Schaefer
361	7Networks_LH_Cont_pCun_1	Cont	Schaefer
362	7Networks_LH_Cont_pCun_2	Cont	Schaefer
363	7Networks_LH_Cont_pCun_3	Cont	Schaefer
364	7Networks_LH_Cont_pCun_4	Cont	Schaefer
365	7Networks_LH_Cont_Cing_1	Cont	Schaefer
366	7Networks_LH_Cont_Cing_2	Cont	Schaefer
367	7Networks_LH_Cont_Cing_3	Cont	Schaefer
368	7Networks_LH_Cont_Cing_4	Cont	Schaefer
369	7Networks_LH_Cont_Cing_5	Cont	Schaefer
370	7Networks_LH_Cont_Cing_6	Cont	Schaefer
371	7Networks_LH_Cont_Cing_7	Cont	Schaefer
372	7Networks_LH_Cont_Cing_8	Cont	Schaefer
373	7Networks_LH_Cont_PFCmp_1	Cont	Schaefer
374	7Networks_LH_Cont_PFCmp_2	Cont	Schaefer
375	7Networks_LH_Default_Par_1	Default	Schaefer
376	7Networks_LH_Default_Par_2	Default	Schaefer
377	7Networks_LH_Default_Par_3	Default	Schaefer
378	7Networks_LH_Default_Par_4	Default	Schaefer
379	7Networks_LH_Default_Par_5	Default	Schaefer
380	7Networks_LH_Default_Par_6	Default	Schaefer
381	7Networks_LH_Default_Par_7	Default	Schaefer
382	7Networks_LH_Default_Par_8	Default	Schaefer
383	7Networks_LH_Default_Par_9	Default	Schaefer
384	7Networks_LH_Default_Par_10	Default	Schaefer
385	7Networks_LH_Default_Par_11	Default	Schaefer
386	7Networks_LH_Default_Par_12	Default	Schaefer
387	7Networks_LH_Default_Par_13	Default	Schaefer
388	7Networks_LH_Default_Par_14	Default	Schaefer
389	7Networks_LH_Default_Par_15	Default	Schaefer
390	7Networks_LH_Default_Par_16	Default	Schaefer
391	7Networks_LH_Default_Par_17	Default	Schaefer
392	7Networks_LH_Default_Par_18	Default	Schaefer
393	7Networks_LH_Default_Par_19	Default	Schaefer
394	7Networks_LH_Default_Temp_1	Default	Schaefer

395	7Networks_LH_Default_Temp_2	Default	Schaefer
396	7Networks_LH_Default_Temp_3	Default	Schaefer
397	7Networks_LH_Default_Temp_4	Default	Schaefer
398	7Networks_LH_Default_Temp_5	Default	Schaefer
399	7Networks_LH_Default_Temp_6	Default	Schaefer
400	7Networks_LH_Default_Temp_7	Default	Schaefer
401	7Networks_LH_Default_Temp_8	Default	Schaefer
402	7Networks_LH_Default_Temp_9	Default	Schaefer
403	7Networks_LH_Default_Temp_10	Default	Schaefer
404	7Networks_LH_Default_Temp_11	Default	Schaefer
405	7Networks_LH_Default_Temp_12	Default	Schaefer
406	7Networks_LH_Default_Temp_13	Default	Schaefer
407	7Networks_LH_Default_Temp_14	Default	Schaefer
408	7Networks_LH_Default_Temp_15	Default	Schaefer
409	7Networks_LH_Default_Temp_16	Default	Schaefer
410	7Networks_LH_Default_Temp_17	Default	Schaefer
411	7Networks_LH_Default_Temp_18	Default	Schaefer
412	7Networks_LH_Default_Temp_19	Default	Schaefer
413	7Networks_LH_Default_Temp_20	Default	Schaefer
414	7Networks_LH_Default_Temp_21	Default	Schaefer
415	7Networks_LH_Default_Temp_22	Default	Schaefer
416	7Networks_LH_Default_PFC_1	Default	Schaefer
417	7Networks_LH_Default_PFC_2	Default	Schaefer
418	7Networks_LH_Default_PFC_3	Default	Schaefer
419	7Networks_LH_Default_PFC_4	Default	Schaefer
420	7Networks_LH_Default_PFC_5	Default	Schaefer
421	7Networks_LH_Default_PFC_6	Default	Schaefer
422	7Networks_LH_Default_PFC_7	Default	Schaefer
423	7Networks_LH_Default_PFC_8	Default	Schaefer
424	7Networks_LH_Default_PFC_9	Default	Schaefer
425	7Networks_LH_Default_PFC_10	Default	Schaefer
426	7Networks_LH_Default_PFC_11	Default	Schaefer
427	7Networks_LH_Default_PFC_12	Default	Schaefer
428	7Networks_LH_Default_PFC_13	Default	Schaefer
429	7Networks_LH_Default_PFC_14	Default	Schaefer
430	7Networks_LH_Default_PFC_15	Default	Schaefer
431	7Networks_LH_Default_PFC_16	Default	Schaefer
432	7Networks_LH_Default_PFC_17	Default	Schaefer
433	7Networks_LH_Default_PFC_18	Default	Schaefer
434	7Networks_LH_Default_PFC_19	Default	Schaefer
435	7Networks_LH_Default_PFC_20	Default	Schaefer
436	7Networks_LH_Default_PFC_21	Default	Schaefer
437	7Networks_LH_Default_PFC_22	Default	Schaefer
438	7Networks_LH_Default_PFC_23	Default	Schaefer

439	7Networks_LH_Default_PFC_24	Default	Schaefer
440	7Networks_LH_Default_PFC_25	Default	Schaefer
441	7Networks_LH_Default_PFC_26	Default	Schaefer
442	7Networks_LH_Default_PFC_27	Default	Schaefer
443	7Networks_LH_Default_PFC_28	Default	Schaefer
444	7Networks_LH_Default_PFC_29	Default	Schaefer
445	7Networks_LH_Default_PFC_30	Default	Schaefer
446	7Networks_LH_Default_PFC_31	Default	Schaefer
447	7Networks_LH_Default_PFC_32	Default	Schaefer
448	7Networks_LH_Default_PFC_33	Default	Schaefer
449	7Networks_LH_Default_PFC_34	Default	Schaefer
450	7Networks_LH_Default_PFC_35	Default	Schaefer
451	7Networks_LH_Default_PFC_36	Default	Schaefer
452	7Networks_LH_Default_PFC_37	Default	Schaefer
453	7Networks_LH_Default_PFC_38	Default	Schaefer
454	7Networks_LH_Default_PFC_39	Default	Schaefer
455	7Networks_LH_Default_PFC_40	Default	Schaefer
456	7Networks_LH_Default_PFC_41	Default	Schaefer
457	7Networks_LH_Default_PFC_42	Default	Schaefer
458	7Networks_LH_Default_PFC_43	Default	Schaefer
459	7Networks_LH_Default_PFC_44	Default	Schaefer
460	7Networks_LH_Default_PFC_45	Default	Schaefer
461	7Networks_LH_Default_PFC_46	Default	Schaefer
462	7Networks_LH_Default_PFC_47	Default	Schaefer
463	7Networks_LH_Default_PFC_48	Default	Schaefer
464	7Networks_LH_Default_PFC_49	Default	Schaefer
465	7Networks_LH_Default_PFC_50	Default	Schaefer
466	7Networks_LH_Default_pCunPCC_1	Default	Schaefer
467	7Networks_LH_Default_pCunPCC_2	Default	Schaefer
468	7Networks_LH_Default_pCunPCC_3	Default	Schaefer
469	7Networks_LH_Default_pCunPCC_4	Default	Schaefer
470	7Networks_LH_Default_pCunPCC_5	Default	Schaefer
471	7Networks_LH_Default_pCunPCC_6	Default	Schaefer
472	7Networks_LH_Default_pCunPCC_7	Default	Schaefer
473	7Networks_LH_Default_pCunPCC_8	Default	Schaefer
474	7Networks_LH_Default_pCunPCC_9	Default	Schaefer
475	7Networks_LH_Default_pCunPCC_10	Default	Schaefer
476	7Networks_LH_Default_pCunPCC_11	Default	Schaefer
477	7Networks_LH_Default_pCunPCC_12	Default	Schaefer
478	7Networks_LH_Default_pCunPCC_13	Default	Schaefer
479	7Networks_LH_Default_pCunPCC_14	Default	Schaefer
480	7Networks_LH_Default_pCunPCC_15	Default	Schaefer
481	7Networks_LH_Default_pCunPCC_16	Default	Schaefer
482	7Networks_LH_Default_pCunPCC_17	Default	Schaefer

483	7Networks_LH_Default_pCunPCC_18	Default	Schaefer
484	7Networks_LH_Default_pCunPCC_19	Default	Schaefer
485	7Networks_LH_Default_pCunPCC_20	Default	Schaefer
486	7Networks_LH_Default_pCunPCC_21	Default	Schaefer
487	7Networks_LH_Default_pCunPCC_22	Default	Schaefer
488	7Networks_LH_Default_pCunPCC_23	Default	Schaefer
489	7Networks_LH_Default_pCunPCC_24	Default	Schaefer
490	7Networks_LH_Default_pCunPCC_25	Default	Schaefer
491	7Networks_LH_Default_pCunPCC_26	Default	Schaefer
492	7Networks_LH_Default_pCunPCC_27	Default	Schaefer
493	7Networks_LH_Default_pCunPCC_28	Default	Schaefer
494	7Networks_LH_Default_pCunPCC_29	Default	Schaefer
495	7Networks_LH_Default_pCunPCC_30	Default	Schaefer
496	7Networks_LH_Default_pCunPCC_31	Default	Schaefer
497	7Networks_LH_Default_pCunPCC_32	Default	Schaefer
498	7Networks_LH_Default_PHC_1	Default	Schaefer
499	7Networks_LH_Default_PHC_2	Default	Schaefer
500	7Networks_LH_Default_PHC_3	Default	Schaefer
501	7Networks_RH_Vis_1	Vis	Schaefer
502	7Networks_RH_Vis_2	Vis	Schaefer
503	7Networks_RH_Vis_3	Vis	Schaefer
504	7Networks_RH_Vis_4	Vis	Schaefer
505	7Networks_RH_Vis_5	Vis	Schaefer
506	7Networks_RH_Vis_6	Vis	Schaefer
507	7Networks_RH_Vis_7	Vis	Schaefer
508	7Networks_RH_Vis_8	Vis	Schaefer
509	7Networks_RH_Vis_9	Vis	Schaefer
510	7Networks_RH_Vis_10	Vis	Schaefer
511	7Networks_RH_Vis_11	Vis	Schaefer
512	7Networks_RH_Vis_12	Vis	Schaefer
513	7Networks_RH_Vis_13	Vis	Schaefer
514	7Networks_RH_Vis_14	Vis	Schaefer
515	7Networks_RH_Vis_15	Vis	Schaefer
516	7Networks_RH_Vis_16	Vis	Schaefer
517	7Networks_RH_Vis_17	Vis	Schaefer
518	7Networks_RH_Vis_18	Vis	Schaefer
519	7Networks_RH_Vis_19	Vis	Schaefer
520	7Networks_RH_Vis_20	Vis	Schaefer
521	7Networks_RH_Vis_21	Vis	Schaefer
522	7Networks_RH_Vis_22	Vis	Schaefer
523	7Networks_RH_Vis_23	Vis	Schaefer
524	7Networks_RH_Vis_24	Vis	Schaefer
525	7Networks_RH_Vis_25	Vis	Schaefer
526	7Networks_RH_Vis_26	Vis	Schaefer

527	7Networks_RH_Vis_27	Vis	Schaefer
528	7Networks_RH_Vis_28	Vis	Schaefer
529	7Networks_RH_Vis_29	Vis	Schaefer
530	7Networks_RH_Vis_30	Vis	Schaefer
531	7Networks_RH_Vis_31	Vis	Schaefer
532	7Networks_RH_Vis_32	Vis	Schaefer
533	7Networks_RH_Vis_33	Vis	Schaefer
534	7Networks_RH_Vis_34	Vis	Schaefer
535	7Networks_RH_Vis_35	Vis	Schaefer
536	7Networks_RH_Vis_36	Vis	Schaefer
537	7Networks_RH_Vis_37	Vis	Schaefer
538	7Networks_RH_Vis_38	Vis	Schaefer
539	7Networks_RH_Vis_39	Vis	Schaefer
540	7Networks_RH_Vis_40	Vis	Schaefer
541	7Networks_RH_Vis_41	Vis	Schaefer
542	7Networks_RH_Vis_42	Vis	Schaefer
543	7Networks_RH_Vis_43	Vis	Schaefer
544	7Networks_RH_Vis_44	Vis	Schaefer
545	7Networks_RH_Vis_45	Vis	Schaefer
546	7Networks_RH_Vis_46	Vis	Schaefer
547	7Networks_RH_Vis_47	Vis	Schaefer
548	7Networks_RH_Vis_48	Vis	Schaefer
549	7Networks_RH_Vis_49	Vis	Schaefer
550	7Networks_RH_Vis_50	Vis	Schaefer
551	7Networks_RH_Vis_51	Vis	Schaefer
552	7Networks_RH_Vis_52	Vis	Schaefer
553	7Networks_RH_Vis_53	Vis	Schaefer
554	7Networks_RH_Vis_54	Vis	Schaefer
555	7Networks_RH_Vis_55	Vis	Schaefer
556	7Networks_RH_Vis_56	Vis	Schaefer
557	7Networks_RH_Vis_57	Vis	Schaefer
558	7Networks_RH_Vis_58	Vis	Schaefer
559	7Networks_RH_Vis_59	Vis	Schaefer
560	7Networks_RH_Vis_60	Vis	Schaefer
561	7Networks_RH_Vis_61	Vis	Schaefer
562	7Networks_RH_Vis_62	Vis	Schaefer
563	7Networks_RH_Vis_63	Vis	Schaefer
564	7Networks_RH_Vis_64	Vis	Schaefer
565	7Networks_RH_Vis_65	Vis	Schaefer
566	7Networks_RH_Vis_66	Vis	Schaefer
567	7Networks_RH_Vis_67	Vis	Schaefer
568	7Networks_RH_Vis_68	Vis	Schaefer
569	7Networks_RH_Vis_69	Vis	Schaefer
570	7Networks_RH_Vis_70	Vis	Schaefer

571	7Networks_RH_Vis_71	Vis	Schaefer
572	7Networks_RH_Vis_72	Vis	Schaefer
573	7Networks_RH_Vis_73	Vis	Schaefer
574	7Networks_RH_Vis_74	Vis	Schaefer
575	7Networks_RH_Vis_75	Vis	Schaefer
576	7Networks_RH_Vis_76	Vis	Schaefer
577	7Networks_RH_Vis_77	Vis	Schaefer
578	7Networks_RH_Vis_78	Vis	Schaefer
579	7Networks_RH_Vis_79	Vis	Schaefer
580	7Networks_RH_Vis_80	Vis	Schaefer
581	7Networks_RH_Vis_81	Vis	Schaefer
582	7Networks_RH_SomMot_1	SomMot	Schaefer
583	7Networks_RH_SomMot_2	SomMot	Schaefer
584	7Networks_RH_SomMot_3	SomMot	Schaefer
585	7Networks_RH_SomMot_4	SomMot	Schaefer
586	7Networks_RH_SomMot_5	SomMot	Schaefer
587	7Networks_RH_SomMot_6	SomMot	Schaefer
588	7Networks_RH_SomMot_7	SomMot	Schaefer
589	7Networks_RH_SomMot_8	SomMot	Schaefer
590	7Networks_RH_SomMot_9	SomMot	Schaefer
591	7Networks_RH_SomMot_10	SomMot	Schaefer
592	7Networks_RH_SomMot_11	SomMot	Schaefer
593	7Networks_RH_SomMot_12	SomMot	Schaefer
594	7Networks_RH_SomMot_13	SomMot	Schaefer
595	7Networks_RH_SomMot_14	SomMot	Schaefer
596	7Networks_RH_SomMot_15	SomMot	Schaefer
597	7Networks_RH_SomMot_16	SomMot	Schaefer
598	7Networks_RH_SomMot_17	SomMot	Schaefer
599	7Networks_RH_SomMot_18	SomMot	Schaefer
600	7Networks_RH_SomMot_19	SomMot	Schaefer
601	7Networks_RH_SomMot_20	SomMot	Schaefer
602	7Networks_RH_SomMot_21	SomMot	Schaefer
603	7Networks_RH_SomMot_22	SomMot	Schaefer
604	7Networks_RH_SomMot_23	SomMot	Schaefer
605	7Networks_RH_SomMot_24	SomMot	Schaefer
606	7Networks_RH_SomMot_25	SomMot	Schaefer
607	7Networks_RH_SomMot_26	SomMot	Schaefer
608	7Networks_RH_SomMot_27	SomMot	Schaefer
609	7Networks_RH_SomMot_28	SomMot	Schaefer
610	7Networks_RH_SomMot_29	SomMot	Schaefer
611	7Networks_RH_SomMot_30	SomMot	Schaefer
612	7Networks_RH_SomMot_31	SomMot	Schaefer
613	7Networks_RH_SomMot_32	SomMot	Schaefer
614	7Networks_RH_SomMot_33	SomMot	Schaefer

615	7Networks_RH_SomMot_34	SomMot	Schaefer
616	7Networks_RH_SomMot_35	SomMot	Schaefer
617	7Networks_RH_SomMot_36	SomMot	Schaefer
618	7Networks_RH_SomMot_37	SomMot	Schaefer
619	7Networks_RH_SomMot_38	SomMot	Schaefer
620	7Networks_RH_SomMot_39	SomMot	Schaefer
621	7Networks_RH_SomMot_40	SomMot	Schaefer
622	7Networks_RH_SomMot_41	SomMot	Schaefer
623	7Networks_RH_SomMot_42	SomMot	Schaefer
624	7Networks_RH_SomMot_43	SomMot	Schaefer
625	7Networks_RH_SomMot_44	SomMot	Schaefer
626	7Networks_RH_SomMot_45	SomMot	Schaefer
627	7Networks_RH_SomMot_46	SomMot	Schaefer
628	7Networks_RH_SomMot_47	SomMot	Schaefer
629	7Networks_RH_SomMot_48	SomMot	Schaefer
630	7Networks_RH_SomMot_49	SomMot	Schaefer
631	7Networks_RH_SomMot_50	SomMot	Schaefer
632	7Networks_RH_SomMot_51	SomMot	Schaefer
633	7Networks_RH_SomMot_52	SomMot	Schaefer
634	7Networks_RH_SomMot_53	SomMot	Schaefer
635	7Networks_RH_SomMot_54	SomMot	Schaefer
636	7Networks_RH_SomMot_55	SomMot	Schaefer
637	7Networks_RH_SomMot_56	SomMot	Schaefer
638	7Networks_RH_SomMot_57	SomMot	Schaefer
639	7Networks_RH_SomMot_58	SomMot	Schaefer
640	7Networks_RH_SomMot_59	SomMot	Schaefer
641	7Networks_RH_SomMot_60	SomMot	Schaefer
642	7Networks_RH_SomMot_61	SomMot	Schaefer
643	7Networks_RH_SomMot_62	SomMot	Schaefer
644	7Networks_RH_SomMot_63	SomMot	Schaefer
645	7Networks_RH_SomMot_64	SomMot	Schaefer
646	7Networks_RH_SomMot_65	SomMot	Schaefer
647	7Networks_RH_SomMot_66	SomMot	Schaefer
648	7Networks_RH_SomMot_67	SomMot	Schaefer
649	7Networks_RH_SomMot_68	SomMot	Schaefer
650	7Networks_RH_SomMot_69	SomMot	Schaefer
651	7Networks_RH_SomMot_70	SomMot	Schaefer
652	7Networks_RH_SomMot_71	SomMot	Schaefer
653	7Networks_RH_SomMot_72	SomMot	Schaefer
654	7Networks_RH_SomMot_73	SomMot	Schaefer
655	7Networks_RH_SomMot_74	SomMot	Schaefer
656	7Networks_RH_SomMot_75	SomMot	Schaefer
657	7Networks_RH_SomMot_76	SomMot	Schaefer
658	7Networks_RH_SomMot_77	SomMot	Schaefer

659	7Networks_RH_SomMot_78	SomMot	Schaefer
660	7Networks_RH_SomMot_79	SomMot	Schaefer
661	7Networks_RH_SomMot_80	SomMot	Schaefer
662	7Networks_RH_SomMot_81	SomMot	Schaefer
663	7Networks_RH_SomMot_82	SomMot	Schaefer
664	7Networks_RH_SomMot_83	SomMot	Schaefer
665	7Networks_RH_SomMot_84	SomMot	Schaefer
666	7Networks_RH_SomMot_85	SomMot	Schaefer
667	7Networks_RH_SomMot_86	SomMot	Schaefer
668	7Networks_RH_SomMot_87	SomMot	Schaefer
669	7Networks_RH_SomMot_88	SomMot	Schaefer
670	7Networks_RH_SomMot_89	SomMot	Schaefer
671	7Networks_RH_SomMot_90	SomMot	Schaefer
672	7Networks_RH_SomMot_91	SomMot	Schaefer
673	7Networks_RH_SomMot_92	SomMot	Schaefer
674	7Networks_RH_SomMot_93	SomMot	Schaefer
675	7Networks_RH_SomMot_94	SomMot	Schaefer
676	7Networks_RH_SomMot_95	SomMot	Schaefer
677	7Networks_RH_SomMot_96	SomMot	Schaefer
678	7Networks_RH_SomMot_97	SomMot	Schaefer
679	7Networks_RH_SomMot_98	SomMot	Schaefer
680	7Networks_RH_SomMot_99	SomMot	Schaefer
681	7Networks_RH_SomMot_100	SomMot	Schaefer
682	7Networks_RH_SomMot_101	SomMot	Schaefer
683	7Networks_RH_SomMot_102	SomMot	Schaefer
684	7Networks_RH_SomMot_103	SomMot	Schaefer
685	7Networks_RH_DorsAttn_Post_1	SalVentAt	Schaefer
686	7Networks_RH_DorsAttn_Post_2	SalVentAt	Schaefer
687	7Networks_RH_DorsAttn_Post_3	SalVentAt	Schaefer
688	7Networks_RH_DorsAttn_Post_4	SalVentAt	Schaefer
689	7Networks_RH_DorsAttn_Post_5	SalVentAt	Schaefer
690	7Networks_RH_DorsAttn_Post_6	SalVentAt	Schaefer
691	7Networks_RH_DorsAttn_Post_7	SalVentAt	Schaefer
692	7Networks_RH_DorsAttn_Post_8	SalVentAt	Schaefer
693	7Networks_RH_DorsAttn_Post_9	SalVentAt	Schaefer
694	7Networks_RH_DorsAttn_Post_10	SalVentAt	Schaefer
695	7Networks_RH_DorsAttn_Post_11	SalVentAt	Schaefer
696	7Networks_RH_DorsAttn_Post_12	SalVentAt	Schaefer
697	7Networks_RH_DorsAttn_Post_13	SalVentAt	Schaefer
698	7Networks_RH_DorsAttn_Post_14	SalVentAt	Schaefer
699	7Networks_RH_DorsAttn_Post_15	SalVentAt	Schaefer
700	7Networks_RH_DorsAttn_Post_16	SalVentAt	Schaefer
701	7Networks_RH_DorsAttn_Post_17	SalVentAt	Schaefer
702	7Networks_RH_DorsAttn_Post_18	SalVentAt	Schaefer

703	7Networks_RH_DorsAttn_Post_19	SalVentAt	Schaefer
704	7Networks_RH_DorsAttn_Post_20	SalVentAt	Schaefer
705	7Networks_RH_DorsAttn_Post_21	SalVentAt	Schaefer
706	7Networks_RH_DorsAttn_Post_22	SalVentAt	Schaefer
707	7Networks_RH_DorsAttn_Post_23	SalVentAt	Schaefer
708	7Networks_RH_DorsAttn_Post_24	SalVentAt	Schaefer
709	7Networks_RH_DorsAttn_Post_25	SalVentAt	Schaefer
710	7Networks_RH_DorsAttn_Post_26	SalVentAt	Schaefer
711	7Networks_RH_DorsAttn_Post_27	SalVentAt	Schaefer
712	7Networks_RH_DorsAttn_Post_28	SalVentAt	Schaefer
713	7Networks_RH_DorsAttn_Post_29	SalVentAt	Schaefer
714	7Networks_RH_DorsAttn_Post_30	SalVentAt	Schaefer
715	7Networks_RH_DorsAttn_Post_31	SalVentAt	Schaefer
716	7Networks_RH_DorsAttn_Post_32	SalVentAt	Schaefer
717	7Networks_RH_DorsAttn_Post_33	SalVentAt	Schaefer
718	7Networks_RH_DorsAttn_Post_34	SalVentAt	Schaefer
719	7Networks_RH_DorsAttn_Post_35	SalVentAt	Schaefer
720	7Networks_RH_DorsAttn_Post_36	SalVentAt	Schaefer
721	7Networks_RH_DorsAttn_Post_37	SalVentAt	Schaefer
722	7Networks_RH_DorsAttn_Post_38	SalVentAt	Schaefer
723	7Networks_RH_DorsAttn_Post_39	SalVentAt	Schaefer
724	7Networks_RH_DorsAttn_Post_40	SalVentAt	Schaefer
725	7Networks_RH_DorsAttn_Post_41	SalVentAt	Schaefer
726	7Networks_RH_DorsAttn_Post_42	SalVentAt	Schaefer
727	7Networks_RH_DorsAttn_Post_43	SalVentAt	Schaefer
728	7Networks_RH_DorsAttn_Post_44	SalVentAt	Schaefer
729	7Networks_RH_DorsAttn_Post_45	SalVentAt	Schaefer
730	7Networks_RH_DorsAttn_Post_46	SalVentAt	Schaefer
731	7Networks_RH_DorsAttn_Post_47	SalVentAt	Schaefer
732	7Networks_RH_DorsAttn_Post_48	SalVentAt	Schaefer
733	7Networks_RH_DorsAttn_Post_49	SalVentAt	Schaefer
734	7Networks_RH_DorsAttn_Post_50	SalVentAt	Schaefer
735	7Networks_RH_DorsAttn_Post_51	SalVentAt	Schaefer
736	7Networks_RH_DorsAttn_Post_52	SalVentAt	Schaefer
737	7Networks_RH_DorsAttn_FEF_1	SalVentAt	Schaefer
738	7Networks_RH_DorsAttn_FEF_2	SalVentAt	Schaefer
739	7Networks_RH_DorsAttn_FEF_3	SalVentAt	Schaefer
740	7Networks_RH_DorsAttn_FEF_4	SalVentAt	Schaefer
741	7Networks_RH_DorsAttn_FEF_5	SalVentAt	Schaefer
742	7Networks_RH_DorsAttn_FEF_6	SalVentAt	Schaefer
743	7Networks_RH_DorsAttn_PrCv_1	SalVentAt	Schaefer
744	7Networks_RH_DorsAttn_PrCv_2	SalVentAt	Schaefer
745	7Networks_RH_DorsAttn_PrCv_3	SalVentAt	Schaefer
746	7Networks_RH_SalVentAttn_TempOccPar_1	DorsAttn	Schaefer

747	7Networks_RH_SalVentAttn_TempOccPar_2	DorsAttn	Schaefer
748	7Networks_RH_SalVentAttn_TempOccPar_3	DorsAttn	Schaefer
749	7Networks_RH_SalVentAttn_TempOccPar_4	DorsAttn	Schaefer
750	7Networks_RH_SalVentAttn_TempOccPar_5	DorsAttn	Schaefer
751	7Networks_RH_SalVentAttn_TempOccPar_6	DorsAttn	Schaefer
752	7Networks_RH_SalVentAttn_TempOccPar_7	DorsAttn	Schaefer
753	7Networks_RH_SalVentAttn_TempOccPar_8	DorsAttn	Schaefer
754	7Networks_RH_SalVentAttn_TempOccPar_9	DorsAttn	Schaefer
755	7Networks_RH_SalVentAttn_TempOccPar_10	DorsAttn	Schaefer
756	7Networks_RH_SalVentAttn_TempOccPar_11	DorsAttn	Schaefer
757	7Networks_RH_SalVentAttn_TempOccPar_12	DorsAttn	Schaefer
758	7Networks_RH_SalVentAttn_TempOccPar_13	DorsAttn	Schaefer
759	7Networks_RH_SalVentAttn_TempOccPar_14	DorsAttn	Schaefer
760	7Networks_RH_SalVentAttn_TempOccPar_15	DorsAttn	Schaefer
761	7Networks_RH_SalVentAttn_TempOccPar_16	DorsAttn	Schaefer
762	7Networks_RH_SalVentAttn_TempOccPar_17	DorsAttn	Schaefer
763	7Networks_RH_SalVentAttn_TempOccPar_18	DorsAttn	Schaefer
764	7Networks_RH_SalVentAttn_PrC_1	DorsAttn	Schaefer
765	7Networks_RH_SalVentAttn_PrC_2	DorsAttn	Schaefer
766	7Networks_RH_SalVentAttn_FrOperIns_1	DorsAttn	Schaefer
767	7Networks_RH_SalVentAttn_FrOperIns_2	DorsAttn	Schaefer
768	7Networks_RH_SalVentAttn_FrOperIns_3	DorsAttn	Schaefer
769	7Networks_RH_SalVentAttn_FrOperIns_4	DorsAttn	Schaefer
770	7Networks_RH_SalVentAttn_FrOperIns_5	DorsAttn	Schaefer
771	7Networks_RH_SalVentAttn_FrOperIns_6	DorsAttn	Schaefer
772	7Networks_RH_SalVentAttn_FrOperIns_7	DorsAttn	Schaefer
773	7Networks_RH_SalVentAttn_FrOperIns_8	DorsAttn	Schaefer
774	7Networks_RH_SalVentAttn_FrOperIns_9	DorsAttn	Schaefer
775	7Networks_RH_SalVentAttn_FrOperIns_10	DorsAttn	Schaefer
776	7Networks_RH_SalVentAttn_FrOperIns_11	DorsAttn	Schaefer
777	7Networks_RH_SalVentAttn_FrOperIns_12	DorsAttn	Schaefer
778	7Networks_RH_SalVentAttn_FrOperIns_13	DorsAttn	Schaefer
779	7Networks_RH_SalVentAttn_FrOperIns_14	DorsAttn	Schaefer
780	7Networks_RH_SalVentAttn_FrOperIns_15	DorsAttn	Schaefer
781	7Networks_RH_SalVentAttn_FrOperIns_16	DorsAttn	Schaefer
782	7Networks_RH_SalVentAttn_FrOperIns_17	DorsAttn	Schaefer
783	7Networks_RH_SalVentAttn_FrOperIns_18	DorsAttn	Schaefer
784	7Networks_RH_SalVentAttn_FrOperIns_19	DorsAttn	Schaefer
785	7Networks_RH_SalVentAttn_FrOperIns_20	DorsAttn	Schaefer
786	7Networks_RH_SalVentAttn_FrOperIns_21	DorsAttn	Schaefer
787	7Networks_RH_SalVentAttn_FrOperIns_22	DorsAttn	Schaefer
788	7Networks_RH_SalVentAttn_FrOperIns_23	DorsAttn	Schaefer
789	7Networks_RH_SalVentAttn_FrOperIns_24	DorsAttn	Schaefer
790	7Networks_RH_SalVentAttn_PFCl_1	DorsAttn	Schaefer

791	7Networks_RH_SalVentAttn_PFCl_2	DorsAttn	Schaefer
792	7Networks_RH_SalVentAttn_Med_1	DorsAttn	Schaefer
793	7Networks_RH_SalVentAttn_Med_2	DorsAttn	Schaefer
794	7Networks_RH_SalVentAttn_Med_3	DorsAttn	Schaefer
795	7Networks_RH_SalVentAttn_Med_4	DorsAttn	Schaefer
796	7Networks_RH_SalVentAttn_Med_5	DorsAttn	Schaefer
797	7Networks_RH_SalVentAttn_Med_6	DorsAttn	Schaefer
798	7Networks_RH_SalVentAttn_Med_7	DorsAttn	Schaefer
799	7Networks_RH_SalVentAttn_Med_8	DorsAttn	Schaefer
800	7Networks_RH_SalVentAttn_Med_9	DorsAttn	Schaefer
801	7Networks_RH_SalVentAttn_Med_10	DorsAttn	Schaefer
802	7Networks_RH_SalVentAttn_Med_11	DorsAttn	Schaefer
803	7Networks_RH_SalVentAttn_Med_12	DorsAttn	Schaefer
804	7Networks_RH_SalVentAttn_Med_13	DorsAttn	Schaefer
805	7Networks_RH_SalVentAttn_Med_14	DorsAttn	Schaefer
806	7Networks_RH_SalVentAttn_Med_15	DorsAttn	Schaefer
807	7Networks_RH_SalVentAttn_Med_16	DorsAttn	Schaefer
808	7Networks_RH_SalVentAttn_Med_17	DorsAttn	Schaefer
809	7Networks_RH_SalVentAttn_Med_18	DorsAttn	Schaefer
810	7Networks_RH_SalVentAttn_Med_19	DorsAttn	Schaefer
811	7Networks_RH_SalVentAttn_Med_20	DorsAttn	Schaefer
812	7Networks_RH_Limbic_OFC_1	Limbic	Schaefer
813	7Networks_RH_Limbic_OFC_2	Limbic	Schaefer
814	7Networks_RH_Limbic_OFC_3	Limbic	Schaefer
815	7Networks_RH_Limbic_OFC_4	Limbic	Schaefer
816	7Networks_RH_Limbic_OFC_5	Limbic	Schaefer
817	7Networks_RH_Limbic_OFC_6	Limbic	Schaefer
818	7Networks_RH_Limbic_OFC_7	Limbic	Schaefer
819	7Networks_RH_Limbic_OFC_8	Limbic	Schaefer
820	7Networks_RH_Limbic_OFC_9	Limbic	Schaefer
821	7Networks_RH_Limbic_OFC_10	Limbic	Schaefer
822	7Networks_RH_Limbic_OFC_11	Limbic	Schaefer
823	7Networks_RH_Limbic_OFC_12	Limbic	Schaefer
824	7Networks_RH_Limbic_OFC_13	Limbic	Schaefer
825	7Networks_RH_Limbic_OFC_14	Limbic	Schaefer
826	7Networks_RH_Limbic_TempPole_1	Limbic	Schaefer
827	7Networks_RH_Limbic_TempPole_2	Limbic	Schaefer
828	7Networks_RH_Limbic_TempPole_3	Limbic	Schaefer
829	7Networks_RH_Limbic_TempPole_4	Limbic	Schaefer
830	7Networks_RH_Limbic_TempPole_5	Limbic	Schaefer
831	7Networks_RH_Limbic_TempPole_6	Limbic	Schaefer
832	7Networks_RH_Limbic_TempPole_7	Limbic	Schaefer
833	7Networks_RH_Limbic_TempPole_8	Limbic	Schaefer
834	7Networks_RH_Limbic_TempPole_9	Limbic	Schaefer

835	7Networks_RH_Limbic_TempPole_10	Limbic	Schaefer
836	7Networks_RH_Limbic_TempPole_11	Limbic	Schaefer
837	7Networks_RH_Limbic_TempPole_12	Limbic	Schaefer
838	7Networks_RH_Limbic_TempPole_13	Limbic	Schaefer
839	7Networks_RH_Limbic_TempPole_14	Limbic	Schaefer
840	7Networks_RH_Limbic_TempPole_15	Limbic	Schaefer
841	7Networks_RH_Limbic_TempPole_16	Limbic	Schaefer
842	7Networks_RH_Limbic_TempPole_17	Limbic	Schaefer
843	7Networks_RH_Cont_Par_1	Cont	Schaefer
844	7Networks_RH_Cont_Par_2	Cont	Schaefer
845	7Networks_RH_Cont_Par_3	Cont	Schaefer
846	7Networks_RH_Cont_Par_4	Cont	Schaefer
847	7Networks_RH_Cont_Par_5	Cont	Schaefer
848	7Networks_RH_Cont_Par_6	Cont	Schaefer
849	7Networks_RH_Cont_Par_7	Cont	Schaefer
850	7Networks_RH_Cont_Par_8	Cont	Schaefer
851	7Networks_RH_Cont_Par_9	Cont	Schaefer
852	7Networks_RH_Cont_Par_10	Cont	Schaefer
853	7Networks_RH_Cont_Par_11	Cont	Schaefer
854	7Networks_RH_Cont_Par_12	Cont	Schaefer
855	7Networks_RH_Cont_Par_13	Cont	Schaefer
856	7Networks_RH_Cont_Par_14	Cont	Schaefer
857	7Networks_RH_Cont_Par_15	Cont	Schaefer
858	7Networks_RH_Cont_Par_16	Cont	Schaefer
859	7Networks_RH_Cont_Par_17	Cont	Schaefer
860	7Networks_RH_Cont_Temp_1	Cont	Schaefer
861	7Networks_RH_Cont_Temp_2	Cont	Schaefer
862	7Networks_RH_Cont_Temp_3	Cont	Schaefer
863	7Networks_RH_Cont_Temp_4	Cont	Schaefer
864	7Networks_RH_Cont_PFCv_1	Cont	Schaefer
865	7Networks_RH_Cont_PFCv_2	Cont	Schaefer
866	7Networks_RH_Cont_PFCl_1	Cont	Schaefer
867	7Networks_RH_Cont_PFCl_2	Cont	Schaefer
868	7Networks_RH_Cont_PFCl_3	Cont	Schaefer
869	7Networks_RH_Cont_PFCl_4	Cont	Schaefer
870	7Networks_RH_Cont_PFCl_5	Cont	Schaefer
871	7Networks_RH_Cont_PFCl_6	Cont	Schaefer
872	7Networks_RH_Cont_PFCl_7	Cont	Schaefer
873	7Networks_RH_Cont_PFCl_8	Cont	Schaefer
874	7Networks_RH_Cont_PFCl_9	Cont	Schaefer
875	7Networks_RH_Cont_PFCl_10	Cont	Schaefer
876	7Networks_RH_Cont_PFCl_11	Cont	Schaefer
877	7Networks_RH_Cont_PFCl_12	Cont	Schaefer
878	7Networks_RH_Cont_PFCl_13	Cont	Schaefer

879	7Networks_RH_Cont_PFCl_14	Cont	Schaefer
880	7Networks_RH_Cont_PFCl_15	Cont	Schaefer
881	7Networks_RH_Cont_PFCl_16	Cont	Schaefer
882	7Networks_RH_Cont_PFCl_17	Cont	Schaefer
883	7Networks_RH_Cont_PFCl_18	Cont	Schaefer
884	7Networks_RH_Cont_PFCl_19	Cont	Schaefer
885	7Networks_RH_Cont_PFCl_20	Cont	Schaefer
886	7Networks_RH_Cont_PFCl_21	Cont	Schaefer
887	7Networks_RH_Cont_PFCl_22	Cont	Schaefer
888	7Networks_RH_Cont_PFCl_23	Cont	Schaefer
889	7Networks_RH_Cont_PFCl_24	Cont	Schaefer
890	7Networks_RH_Cont_PFCl_25	Cont	Schaefer
891	7Networks_RH_Cont_PFCl_26	Cont	Schaefer
892	7Networks_RH_Cont_PFCl_27	Cont	Schaefer
893	7Networks_RH_Cont_PFCl_28	Cont	Schaefer
894	7Networks_RH_Cont_PFCl_29	Cont	Schaefer
895	7Networks_RH_Cont_PFCl_30	Cont	Schaefer
896	7Networks_RH_Cont_PFCl_31	Cont	Schaefer
897	7Networks_RH_Cont_PFCl_32	Cont	Schaefer
898	7Networks_RH_Cont_PFCl_33	Cont	Schaefer
899	7Networks_RH_Cont_PFCl_34	Cont	Schaefer
900	7Networks_RH_Cont_pCun_1	Cont	Schaefer
901	7Networks_RH_Cont_pCun_3	Cont	Schaefer
902	7Networks_RH_Cont_pCun_5	Cont	Schaefer
903	7Networks_RH_Cont_Cing_1	Cont	Schaefer
904	7Networks_RH_Cont_Cing_2	Cont	Schaefer
905	7Networks_RH_Cont_Cing_3	Cont	Schaefer
906	7Networks_RH_Cont_Cing_4	Cont	Schaefer
907	7Networks_RH_Cont_Cing_5	Cont	Schaefer
908	7Networks_RH_Cont_PFCmp_1	Cont	Schaefer
909	7Networks_RH_Cont_PFCmp_2	Cont	Schaefer
910	7Networks_RH_Cont_PFCmp_3	Cont	Schaefer
911	7Networks_RH_Cont_PFCmp_4	Cont	Schaefer
912	7Networks_RH_Cont_PFCmp_5	Cont	Schaefer
913	7Networks_RH_Default_Par_1	Default	Schaefer
914	7Networks_RH_Default_Par_2	Default	Schaefer
915	7Networks_RH_Default_Par_3	Default	Schaefer
916	7Networks_RH_Default_Par_4	Default	Schaefer
917	7Networks_RH_Default_Par_5	Default	Schaefer
918	7Networks_RH_Default_Par_6	Default	Schaefer
919	7Networks_RH_Default_Par_7	Default	Schaefer
920	7Networks_RH_Default_Par_8	Default	Schaefer
921	7Networks_RH_Default_Par_9	Default	Schaefer
922	7Networks_RH_Default_Par_10	Default	Schaefer

923	7Networks_RH_Default_Par_11	Default	Schaefer
924	7Networks_RH_Default_Par_12	Default	Schaefer
925	7Networks_RH_Default_Par_13	Default	Schaefer
926	7Networks_RH_Default_Par_14	Default	Schaefer
927	7Networks_RH_Default_Par_15	Default	Schaefer
928	7Networks_RH_Default_Par_16	Default	Schaefer
929	7Networks_RH_Default_Temp_1	Default	Schaefer
930	7Networks_RH_Default_Temp_2	Default	Schaefer
931	7Networks_RH_Default_Temp_3	Default	Schaefer
932	7Networks_RH_Default_Temp_4	Default	Schaefer
933	7Networks_RH_Default_Temp_5	Default	Schaefer
934	7Networks_RH_Default_Temp_6	Default	Schaefer
935	7Networks_RH_Default_Temp_7	Default	Schaefer
936	7Networks_RH_Default_Temp_8	Default	Schaefer
937	7Networks_RH_Default_Temp_9	Default	Schaefer
938	7Networks_RH_Default_Temp_10	Default	Schaefer
939	7Networks_RH_Default_Temp_11	Default	Schaefer
940	7Networks_RH_Default_Temp_12	Default	Schaefer
941	7Networks_RH_Default_Temp_13	Default	Schaefer
942	7Networks_RH_Default_Temp_14	Default	Schaefer
943	7Networks_RH_Default_Temp_15	Default	Schaefer
944	7Networks_RH_Default_Temp_16	Default	Schaefer
945	7Networks_RH_Default_Temp_17	Default	Schaefer
946	7Networks_RH_Default_Temp_18	Default	Schaefer
947	7Networks_RH_Default_PFCv_1	Default	Schaefer
948	7Networks_RH_Default_PFCv_2	Default	Schaefer
949	7Networks_RH_Default_PFCv_3	Default	Schaefer
950	7Networks_RH_Default_PFCv_4	Default	Schaefer
951	7Networks_RH_Default_PFCv_5	Default	Schaefer
952	7Networks_RH_Default_PFCv_6	Default	Schaefer
953	7Networks_RH_Default_PFCv_7	Default	Schaefer
954	7Networks_RH_Default_PFCv_8	Default	Schaefer
955	7Networks_RH_Default_PFCv_9	Default	Schaefer
956	7Networks_RH_Default_PFCv_10	Default	Schaefer
957	7Networks_RH_Default_PFCdPFCm_1	Default	Schaefer
958	7Networks_RH_Default_PFCdPFCm_2	Default	Schaefer
959	7Networks_RH_Default_PFCdPFCm_3	Default	Schaefer
960	7Networks_RH_Default_PFCdPFCm_4	Default	Schaefer
961	7Networks_RH_Default_PFCdPFCm_5	Default	Schaefer
962	7Networks_RH_Default_PFCdPFCm_6	Default	Schaefer
963	7Networks_RH_Default_PFCdPFCm_7	Default	Schaefer
964	7Networks_RH_Default_PFCdPFCm_8	Default	Schaefer
965	7Networks_RH_Default_PFCdPFCm_9	Default	Schaefer
966	7Networks_RH_Default_PFCdPFCm_10	Default	Schaefer

967	7Networks_RH_Default_PFCdPFCm_11	Default	Schaefer
968	7Networks_RH_Default_PFCdPFCm_12	Default	Schaefer
969	7Networks_RH_Default_PFCdPFCm_13	Default	Schaefer
970	7Networks_RH_Default_PFCdPFCm_14	Default	Schaefer
971	7Networks_RH_Default_PFCdPFCm_15	Default	Schaefer
972	7Networks_RH_Default_PFCdPFCm_16	Default	Schaefer
973	7Networks_RH_Default_PFCdPFCm_17	Default	Schaefer
974	7Networks_RH_Default_PFCdPFCm_18	Default	Schaefer
975	7Networks_RH_Default_PFCdPFCm_19	Default	Schaefer
976	7Networks_RH_Default_PFCdPFCm_20	Default	Schaefer
977	7Networks_RH_Default_PFCdPFCm_21	Default	Schaefer
978	7Networks_RH_Default_PFCdPFCm_22	Default	Schaefer
979	7Networks_RH_Default_PFCdPFCm_23	Default	Schaefer
980	7Networks_RH_Default_PFCdPFCm_24	Default	Schaefer
981	7Networks_RH_Default_pCunPCC_1	Default	Schaefer
982	7Networks_RH_Default_pCunPCC_2	Default	Schaefer
983	7Networks_RH_Default_pCunPCC_3	Default	Schaefer
984	7Networks_RH_Default_pCunPCC_4	Default	Schaefer
985	7Networks_RH_Default_pCunPCC_5	Default	Schaefer
986	7Networks_RH_Default_pCunPCC_6	Default	Schaefer
987	7Networks_RH_Default_pCunPCC_7	Default	Schaefer
988	7Networks_RH_Default_pCunPCC_8	Default	Schaefer
989	7Networks_RH_Default_pCunPCC_9	Default	Schaefer
990	7Networks_RH_Default_pCunPCC_10	Default	Schaefer
991	7Networks_RH_Default_pCunPCC_11	Default	Schaefer
992	7Networks_RH_Default_pCunPCC_12	Default	Schaefer
993	7Networks_RH_Default_pCunPCC_13	Default	Schaefer
994	7Networks_RH_Default_pCunPCC_14	Default	Schaefer
995	7Networks_RH_Default_pCunPCC_15	Default	Schaefer
996	7Networks_RH_Default_pCunPCC_16	Default	Schaefer
997	7Networks_RH_Default_pCunPCC_17	Default	Schaefer
998	7Networks_RH_Default_pCunPCC_18	Default	Schaefer
999	7Networks_RH_Cont_pCun_2	Cont	Schaefer
1000	7Networks_RH_Cont_pCun_4	Cont	Schaefer
1001	7Networks_LH_Vis	Vis	Buckner
1002	7Networks_LH_SomMot	SomMot	Buckner
1003	7Networks_LH_SalVentAt	SalVentAt	Buckner
1004	7Networks_LH_DorsAttn	DorsAttn	Buckner
1005	7Networks_LH_Limbic	Limbic	Buckner
1006	7Networks_LH_Cont	Cont	Buckner
1007	7Networks_LH_Default	Default	Buckner
1008	7Networks_RH_Vis	Vis	Buckner
1009	7Networks_RH_SomMot	SomMot	Buckner
1010	7Networks_RH_SalVentAttn	SalVentAt	Buckner

1011	7Networks_RH_DorsAttn	DorsAttn	Buckner
1012	7Networks_RH_Limbic	Limbic	Buckner
1013	7Networks_RH_Cont	Cont	Buckner
1014	7Networks_RH_Default	Default	Buckner
1015	7Networks_LH_SomMot	SomMot	Choi
1016	7Networks_LH_DorsAttn	DorsAttn	Choi
1017	7Networks_LH_Limbic	Limbic	Choi
1018	7Networks_LH_Cont	Cont	Choi
1019	7Networks_LH_Default	Default	Choi
1020	7Networks_RH_SomMot	SomMot	Choi
1021	7Networks_RH_SalVentAttn	SalVentAt	Choi
1022	7Networks_RH_DorsAttn	DorsAttn	Choi
1023	7Networks_RH_Limbic	Limbic	Choi
1024	7Networks_RH_Cont	Cont	Choi
1025	7Networks_RH_Default	Default	Choi
1026	7Networks_LH_Vis	Vis	Thalamic
1027	7Networks_LH_SomMot	SomMot	Thalamic
1028	7Networks_LH_SalVentAt	SalVentAt	Thalamic
1029	7Networks_LH_DorsAttn	DorsAttn	Thalamic
1030	7Networks_LH_Limbic	Limbic	Thalamic
1031	7Networks_LH_Cont	Cont	Thalamic
1032	7Networks_LH_Default	Default	Thalamic
1033	7Networks_RH_Vis	Vis	Thalamic
1034	7Networks_RH_SomMot	SomMot	Thalamic
1035	7Networks_RH_SalVentAttn	SalVentAt	Thalamic
1036	7Networks_RH_DorsAttn	DorsAttn	Thalamic
1037	7Networks_RH_Limbic	Limbic	Thalamic
1038	7Networks_RH_Cont	Cont	Thalamic
1039	7Networks_RH_Default	Default	Thalamic

Table S2 Top 20 KEGG terms for tinnitus-associated Gradient-1 alteration features

Gene Set	Description	Size	Leading Edge Number	ES	*NES*	*P* value	*FDR q* value
hsa03010	Ribosome	130	75	0.53086	2.6151	0	0
hsa03040	Spliceosome	127	56	0.45853	2.2781	0	0
hsa05416	Viral myocarditis	58	30	-0.53457	-2.2771	0	0
hsa04940	Type I diabetes mellitus	43	24	-0.58369	-2.3380	0	0
hsa05150	Staphylococcus aureus infection	51	31	-0.64115	-2.6044	0	0
hsa05330	Allograft rejection	37	20	-0.57024	-2.2135	0	0.00016435
hsa04514	Cell adhesion molecules (CAMs)	142	66	-0.45288	-2.2475	0	0.00019722
hsa05332	Graft-versus-host disease	39	20	-0.58128	-2.2642	0	0.00024653
hsa04640	Hematopoietic cell lineage	94	34	-0.47053	-2.1529	0	0.00042262
hsa05310	Asthma	30	16	-0.56648	-2.0675	0	0.00098610
hsa04612	Antigen processing and presentation	69	41	-0.48046	-2.0709	0	0.00098610
hsa01212	Fatty acid metabolism	47	24	-0.50814	-2.0327	0	0.0010847
hsa03013	RNA transport	149	45	0.37074	1.8723	0	0.038277
hsa04720	Long-term potentiation	67	36	0.40028	1.7487	0	0.10772
hsa05014	Amyotrophic lateral sclerosis (ALS)	51	19	0.40651	1.6763	0.0065217	0.16178
hsa05031	Amphetamine addiction	68	18	0.36962	1.6062	0.0022124	0.20600
hsa04136	Autophagy	31	15	0.41483	1.5294	0.027778	0.22170
hsa04744	Phototransduction	28	14	0.45478	1.6141	0.014957	0.22424
hsa05214	Glioma	70	26	0.33277	1.4706	0.015873	0.22830
hsa04728	Dopaminergic synapse	130	48	0.30281	1.4842	0.0043197	0.23074

Table S3 Top 20 GO BP terms for tinnitus-associated Gradient-1 alteration features

Gene Set	Description	Size	Leading Edge Number	ES	*NES*	*P* value	*FDR q* value
GO:0070972	protein localization to endoplasmic reticulum	135	80	0.48080	2.3792	0	0
GO:0006413	translational initiation	182	94	0.43991	2.2875	0	0.00040042
GO:0043062	extracellular structure organization	395	167	-0.38219	-2.1313	0	0.0081385
GO:0060840	artery development	83	42	-0.43660	-1.9369	0	0.012402
GO:0003158	endothelium development	117	58	-0.39986	-1.9164	0	0.012692
GO:0034341	response to interferon-gamma	184	74	-0.37809	-1.9400	0	0.013225
GO:0051180	vitamin transport	37	16	-0.51074	-1.9265	0	0.013254
GO:0002507	tolerance induction	23	14	-0.59415	-1.9468	0	0.013287
GO:0006929	substrate-dependent cell migration	28	11	-0.54887	-1.9175	0	0.013635
GO:0060485	mesenchyme development	258	79	-0.36313	-1.9502	0	0.015114
GO:0034340	response to type I interferon	84	45	-0.41328	-1.8715	0	0.015579
GO:0003170	heart valve development	55	17	-0.45661	-1.8805	0.0018450	0.015830
GO:0002181	cytoplasmic translation	88	38	0.40795	1.9141	0	0.020662
GO:0008380	RNA splicing	403	174	0.33623	1.9299	0	0.021022
GO:0006397	mRNA processing	466	177	0.33214	1.9387	0	0.025894
GO:0022613	ribonucleoprotein complex biogenesis	425	170	0.32452	1.8642	0	0.027114
GO:0090150	establishment of protein localization to membrane	308	125	0.33101	1.8644	0	0.031633
GO:0008356	asymmetric cell division	19	8	0.55788	1.8126	0.0020080	0.039241
GO:0016072	rRNA metabolic process	233	94	0.32593	1.7717	0	0.043886
GO:0006401	RNA catabolic process	334	136	0.31895	1.7812	0	0.045559

Table S4 Top 20 GO CC terms for tinnitus-associated Gradient-1 alteration features

Gene Set	Description	Size	Leading Edge Number	ES	*NES*	*P* value	*FDR q* value
GO:0005681	spliceosomal complex	169	104	0.45166	2.3573	0	0
GO:0005840	ribosome	227	104	0.42050	2.2753	0	0
GO:0042611	MHC protein complex	21	17	-0.71870	-2.3590	0	0
GO:0044445	cytosolic part	236	118	0.39134	2.1119	0	0.00039183
GO:0005811	lipid droplet	77	37	-0.48463	-2.1617	0	0.00085636
GO:0099572	postsynaptic specialization	334	129	0.36546	2.0367	0	0.0011755
GO:0098984	neuron to neuron synapse	335	129	0.35621	1.9852	0	0.0023510
GO:0030667	secretory granule membrane	285	114	-0.36979	-1.9925	0	0.0025691
GO:0005775	vacuolar lumen	166	54	-0.38666	-1.9435	0	0.0034255
GO:1903293	phosphatase complex	47	26	0.47044	1.8911	0	0.0062693
GO:0120114	Sm-like protein family complex	69	41	0.42147	1.8776	0.0022272	0.0065492
GO:0005844	polysome	73	23	0.41538	1.8655	0	0.0067591
GO:0098636	protein complex involved in cell adhesion	35	21	-0.52048	-1.8837	0	0.0070222
GO:0031012	extracellular matrix	484	176	-0.32743	-1.8573	0	0.0078500
GO:0005581	collagen trimer	85	36	-0.41298	-1.8381	0	0.0080743
GO:0044309	neuron spine	165	50	0.34779	1.8060	0	0.010188
GO:0005796	Golgi lumen	93	46	-0.38868	-1.7835	0	0.012310
GO:0030139	endocytic vesicle	283	108	-0.32389	-1.7398	0	0.016461
GO:0098978	glutamatergic synapse	347	156	0.30853	1.7446	0	0.018338
GO:0016323	basolateral plasma membrane	216	71	-0.32406	-1.6843	0	0.018511

Table S5 Top 20 GO MF terms for tinnitus-associated Gradient-1 alteration features

Gene Set	Description	Size	Leading Edge Number	ES	*NES*	*P* value	*FDR q* value
GO:0003735	structural constituent of ribosome	154	81	0.47794	2.4447	0	0
GO:0019843	rRNA binding	62	32	0.48557	2.1459	0	0.0034693
GO:0005518	collagen binding	67	31	-0.49014	-2.1107	0	0.0060444
GO:0005201	extracellular matrix structural constituent	157	65	-0.40189	-2.0137	0	0.011081
GO:0030332	cyclin binding	30	15	0.54108	1.9688	0.0021097	0.014538
GO:0005496	steroid binding	90	38	-0.42848	-1.9372	0	0.017797
GO:0090482	vitamin transmembrane transporter activity	26	10	-0.53239	-1.8243	0.0057582	0.029886
GO:0005319	lipid transporter activity	143	71	-0.37020	-1.8266	0	0.034655
GO:0004896	cytokine receptor activity	92	36	-0.41020	-1.8431	0	0.036518
GO:0050840	extracellular matrix binding	54	16	-0.42651	-1.7507	0.0019802	0.053392
GO:0016298	lipase activity	129	46	-0.35776	-1.7343	0	0.055155
GO:0003823	antigen binding	55	17	-0.41841	-1.7181	0.0036969	0.056303
GO:0051184	cofactor transmembrane transporter activity	22	11	-0.49985	-1.6506	0.018416	0.065697
GO:0008135	translation factor activity, RNA binding	83	28	0.37874	1.7102	0	0.13258
GO:0031490	chromatin DNA binding	103	41	0.33278	1.5995	0.0044248	0.18833
GO:0036002	pre-mRNA binding	32	13	0.42382	1.6098	0.010526	0.20436
GO:0003729	mRNA binding	226	82	0.30166	1.6176	0	0.23096
GO:0005516	calmodulin binding	192	43	0.27727	1.4504	0.0045977	0.26076
GO:0019208	phosphatase regulator activity	88	39	0.31163	1.4388	0.015521	0.26491
GO:0048156	tau protein binding	45	18	0.36243	1.4578	0.044625	0.26544

Table S6 Top 20 KEGG terms for tinnitus-associated Gradient-2 alteration features

Gene Set	Description	Size	Leading Edge Number	ES	*NES*	*P* value	*FDR q* value
hsa00190	Oxidative phosphorylation	118	81	0.56073	2.3607	0	0
hsa05310	Asthma	30	16	-0.60097	-2.3086	0	0
hsa04940	Type I diabetes mellitus	43	20	-0.55163	-2.3609	0	0
hsa05332	Graft-versus-host disease	39	19	-0.58933	-2.4103	0	0
hsa05320	Autoimmune thyroid disease	46	20	-0.56080	-2.4530	0	0
hsa05330	Allograft rejection	37	19	-0.60198	-2.4805	0	0
hsa05150	Staphylococcus aureus infection	51	31	-0.59044	-2.5299	0	0
hsa05322	Systemic lupus erythematosus	114	65	-0.52697	-2.6462	0	0
hsa04514	Cell adhesion molecules (CAMs)	142	64	-0.41459	-2.2287	0	0.00025619
hsa04672	Intestinal immune network for IgA production	47	20	-0.49397	-2.1631	0	0.00079704
hsa04640	Hematopoietic cell lineage	94	41	-0.42214	-2.0992	0	0.0013322
hsa04723	Retrograde endocannabinoid signaling	140	76	0.47340	2.0352	0	0.0029770
hsa05012	Parkinson disease	125	78	0.46069	1.9434	0	0.0064501
hsa04932	Non-alcoholic fatty liver disease (NAFLD)	143	55	0.43777	1.9119	0	0.0068046
hsa04714	Thermogenesis	212	110	0.43083	1.9537	0	0.0072771
hsa05016	Huntington disease	183	108	0.43207	1.9137	0	0.0079386
hsa04140	Autophagy	126	59	0.44634	1.9203	0	0.0091294
hsa04370	VEGF signaling pathway	56	30	0.49103	1.8507	0	0.011357
hsa05010	Alzheimer disease	163	74	0.42320	1.8603	0	0.011536
hsa04966	Collecting duct acid secretion	27	13	0.55703	1.8290	0.0028653	0.013198

Table S7 Top 20 GO BP terms for tinnitus-associated Gradient-2 alteration features

Gene Set	Description	Size	Leading Edge Number	ES	*NES*	*P* value	*FDR q* value
GO:1903008	organelle disassembly	96	55	0.54663	2.2700	0	0
GO:0033108	mitochondrial respiratory chain complex assembly	88	51	0.52752	2.1665	0	0.00041036
GO:0010257	NADH dehydrogenase complex assembly	58	41	0.55852	2.1138	0	0.00054714
GO:0035418	protein localization to synapse	61	33	0.54258	2.0577	0	0.0016414
GO:0099003	vesicle-mediated transport in synapse	199	107	0.45559	2.0470	0	0.0018056
GO:0099177	regulation of trans-synaptic signaling	414	179	0.41768	1.9982	0	0.0036932
GO:0000002	mitochondrial genome maintenance	29	15	0.60389	1.9849	0	0.0038691
GO:0072512	trivalent inorganic cation transport	38	20	0.57012	1.9657	0	0.0045139
GO:0051648	vesicle localization	299	127	0.41383	1.9210	0	0.0054465
GO:0002209	behavioral defense response	37	18	0.55635	1.9217	0	0.0059912
GO:1990868	response to chemokine	89	44	-0.44417	-2.1467	0	0.012945
GO:0032609	interferon-gamma production	105	35	-0.33684	-1.6891	0	0.15302
GO:0050900	leukocyte migration	408	130	-0.27434	-1.6365	0	0.15657
GO:0043062	extracellular structure organization	395	149	-0.28378	-1.6660	0	0.16273
GO:0060037	pharyngeal system development	26	12	-0.45295	-1.6407	0.011730	0.16357
GO:0048483	autonomic nervous system development	46	17	-0.40505	-1.7089	0	0.17321
GO:0050866	negative regulation of cell activation	177	61	-0.30402	-1.6897	0	0.17383
GO:0002507	tolerance induction	23	14	-0.52121	-1.8509	0	0.17752
GO:0036314	response to sterol	28	15	-0.43197	-1.6087	0.013158	0.17824
GO:0070661	leukocyte proliferation	273	80	-0.28268	-1.6414	0	0.17937

Table S8 Top 20 GO CC terms for tinnitus-associated Gradient-2 alteration features

Gene Set	Description	Size	Leading Edge Number	ES	*NES*	*P* value	*FDR q* value
GO:0044455	mitochondrial membrane part	209	127	0.53251	2.4008	0	0
GO:0070469	respiratory chain	88	46	0.57261	2.3464	0	0
GO:0098798	mitochondrial protein complex	249	125	0.49048	2.2562	0	0
GO:0098685	Schaffer collateral - CA1 synapse	81	41	0.56300	2.2538	0	0
GO:0030964	NADH dehydrogenase complex	45	34	0.60926	2.2066	0	0
GO:0099572	postsynaptic specialization	334	166	0.46205	2.1898	0	0
GO:0044815	DNA packaging complex	66	26	-0.52240	-2.4260	0	0
GO:0042611	MHC protein complex	21	17	-0.72328	-2.4911	0	0
GO:1990204	oxidoreductase complex	98	58	0.50704	2.1004	0	0.000094436
GO:0098978	glutamatergic synapse	347	158	0.45456	2.1399	0	0.00010493
GO:0005743	mitochondrial inner membrane	431	218	0.45182	2.1649	0	0.00011805
GO:0098984	neuron to neuron synapse	335	168	0.46098	2.1748	0	0.00013491
GO:0098636	protein complex involved in cell adhesion	35	14	-0.46632	-1.8196	0.0065359	0.027310
GO:0032993	protein-DNA complex	151	50	-0.33823	-1.7672	0	0.032585
GO:0030667	secretory granule membrane	285	88	-0.26646	-1.5172	0	0.12372
GO:0005796	Golgi lumen	93	34	-0.31311	-1.5275	0.0050761	0.13978
GO:0098552	side of membrane	476	128	-0.24034	-1.4478	0	0.17379
GO:0005775	vacuolar lumen	166	47	-0.25865	-1.3977	0	0.20886
GO:0031012	extracellular matrix	484	160	-0.21380	-1.2959	0	0.31605
GO:0005790	smooth endoplasmic reticulum	34	17	-0.33827	-1.2963	0.11285	0.35061

Table S9 Top 20 GO MF terms for tinnitus-associated Gradient-2 alteration features

Gene Set	Description	Size	Leading Edge Number	ES	*NES*	*P* value	*FDR q* value
GO:0038187	pattern recognition receptor activity	20	14	-0.58383	-2.0219	0	0.016367
GO:0019955	cytokine binding	125	47	-0.35938	-1.8671	0	0.025823
GO:0004896	cytokine receptor activity	92	47	-0.36781	-1.8038	0	0.029679
GO:0003823	antigen binding	55	28	-0.40930	-1.8132	0	0.033825
GO:0005518	collagen binding	67	36	-0.40640	-1.8701	0	0.037644
GO:0030332	cyclin binding	30	15	0.52306	1.7333	0.0087209	0.10589
GO:0016769	transferase activity, transferring nitrogenous groups	23	8	0.54327	1.7030	0.0090361	0.11450
GO:0017137	Rab GTPase binding	172	57	0.36121	1.6095	0	0.11459
GO:0019843	rRNA binding	62	25	0.41749	1.6015	0.0012970	0.11531
GO:0019787	ubiquitin-like protein transferase activity	405	152	0.34061	1.6160	0	0.11676
GO:0008022	protein C-terminus binding	183	83	0.35313	1.5816	0.0011223	0.12165
GO:0016247	channel regulator activity	132	52	0.41310	1.7804	0	0.12219
GO:0004712	protein serine/threonine/tyrosine kinase activity	43	23	0.44237	1.5731	0.011268	0.12354
GO:0000149	SNARE binding	101	49	0.39237	1.6182	0.0012407	0.12472
GO:0015631	tubulin binding	321	120	0.33807	1.5860	0	0.12476
GO:0015026	coreceptor activity	42	12	-0.38544	-1.5965	0.0036630	0.13639
GO:1990841	promoter-specific chromatin binding	45	16	-0.37271	-1.5725	0.021505	0.14029
GO:0019865	immunoglobulin binding	21	11	-0.45016	-1.5414	0.032544	0.15262
GO:0005201	extracellular matrix structural constituent	157	53	-0.26689	-1.4490	0	0.25520
GO:1901618	organic hydroxy compound transmembrane transporter activity	43	15	-0.33728	-1.4039	0.033088	0.30977
